# Anomaly Detection for Structural and Functional Connectivity in Glioma Patients

**DOI:** 10.1002/nbm.70238

**Published:** 2026-03-10

**Authors:** Maria Colpo, Ryan Pollitt, Alexander Leemans, Diego Cecchin, Maurizio Corbetta, Alessandra Bertoldo, Alberto De Luca

**Affiliations:** ^1^ Padova Neuroscience Center University of Padova Padova Italy; ^2^ Department of Information Engineering University of Padova Padova Italy; ^3^ Image Sciences Institute University Medical Center Utrecht Utrecht the Netherlands; ^4^ Department of Medicine, Unit of Nuclear Medicine University of Padova Padova Italy; ^5^ Department of Neuroscience University of Padova Padova Italy; ^6^ Neurology Department, UMC Utrecht Brain Center University Medical Center Utrecht Utrecht the Netherlands

**Keywords:** anomaly detection, brain tumor, functional connectivity, glioma, integration, single subject, structural connectivity, variational autoencoder

## Abstract

Brain connectivity, quantified with diffusion MRI (structural connectivity, SC) and resting‐state functional MRI (functional connectivity, FC), can offer crucial insights into glioma‐brain network interactions. Currently, no standardized approach exists to integrate information from FC and SC and to identify potential tumor‐induced abnormalities at the single‐patient level. Variational autoencoders (VAEs) have been shown to be promising for learning the distribution of features representing a healthy brain and deviations thereof and can naturally be applicable to multiple modalities. This study explores the potential of VAE to integrate FC and SC and detect multimodal anomalies in brain connectivity in glioma patients. The VAE is trained on concatenated FC‐SC healthy data to learn how to reconstruct normative connectivity patterns. After ad hoc transfer learning, the model parameters are applied to the oncological dataset, to obtain the healthy version of the pathological matrices. Given the healthy, pathological, and reconstructed matrices, a statistic is developed with the goal of identifying specific alterations in SC, FC, and their FC + SC integration in glioma patients. SC, FC, and FC + SC abnormalities are compared with each other to explore their interplay and their link with tumor and surrounding brain tissues. Results show that FC is more sensitive to alterations distant from the tumor, while SC is more affected in its vicinity. Then, the alterations identified by FC are generally more in agreement with the alterations identified by FC + SC compared with those highlighted by SC. Moreover, SC abnormalities never overlap with FC + SC out of the tumor, and FC and SC single impairments partially overlap within the tumor core and never overlie in other brain tissues. This information could facilitate patient stratification, prognostic modeling, and personalized treatment planning.

## Graphical abstract

Structural connectivity (SC) and functional connectivity (FC) provide crucial insights into glioma‐brain interactions. This study uses variational autoencoder to integrate FC and SC, detect anomalies, and investigate connectivity impairments in networks proximal and distal to the tumor.

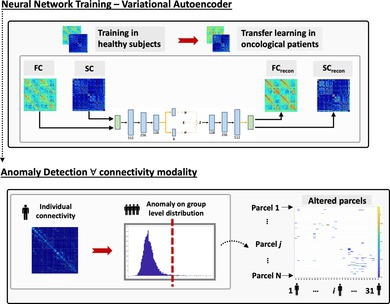

**Abbreviations**
ANTs: Advanced Normalization Tools
BOLD: blood oxygenation level dependent
ControlA: Control‐A network
ControlB: Control‐B network
ControlC: Control‐C network
DefaultA: Default‐A network
DefaultB: Default‐B network
DefaultC: Default‐C network
DMN: Default Mode Network
dMRI: diffusion MRI
DorsAttnA: Dorsal Attention‐A network
DorsAttnB: Dorsal Attention‐B network
DWI: diffusion weighted images
EPI: echoplanar imaging
FA: fractional anisotropy
FC: functional connectivity
FC_recon_
: FC reconstructed
fMRI: functional MRI
GM: gray matter
GD: global disruption
HCP: Human Connectome Project
HCP‐A: HCP‐Aging
IRB: Institutional Review Board
Leaky ReLU: leaky rectified linear unit
LimbicA: Limbic‐A network
LimbicB: Limbic‐B network
NAD: network alteration degree
NOS: number of streamlines
O: edema
OI: overlap index
OS: overall survival
PCA: principal component analysis
ROI: region of interest
rs‐fMRI: resting‐state functional MRI
SalVentAttnA: Salience/Ventral Attention‐A network
SalVentAttB: Salience/Ventral Attention‐B network
SC: structural connectivity
SC_recon_
: SC reconstructed
SomMotA: Somatomotor‐A network
SomMotB: Somatomotor‐B network
T1w: T1‐weighted image
T2w: T2‐weighted image
T: tumor mask
T + O: tumor and edema area mask
TE: echo time
TempPar: Temporal Parietal network
TR: repetition time
t‐SNE: t‐distributed stochastic neighbor embedding
VAE: variational autoencoder
VisCent: Visual Central network
VisPeri: Visual Peripheral network
WM: white matter
zFC: z‐Fisher functional connectivity
zSC: z‐Fisher structural connectivity

## 1 Introduction

The brain is structured in regions of electrically active and interconnected neurons and glial cells, organized in communicating (sub)networks with different degrees of specialization. Brain connectivity aims to model such complex networks by looking at their anatomical or functional coupling markers [1].

In neuro‐oncology, brain connectivity has been defined as influential and critical, marking a shift from a localized approach to a recognized need for understanding the interaction between the tumor and brain networks [2]. This is particularly relevant in the case of glioma, which is the most frequent group of primary brain tumors in the adult population [3, 4, 5]. Typically, the primary brain lesion migrates through the central nervous system, leading first to a functional deterioration and eventually to death [4, 6, 7]. A characteristic of gliomas is their aggressive behavior in infiltrating adjacent tissues [8]. Furthermore, gliomas can affect the white matter (WM) axonal integrity [9] and brain functionalities [10, 11] through invasion, expansion, and intratumoral changes [12]. As such, connectivity can be affected by gliomas, which have been shown to disrupt and alter existing brain networks [13]. In particular, neuron‐tumor synapses formed by glioma cells integrate into already existing brain networks through their electrically active organization. These synaptic connections enable direct neuroglioma communication, allowing tumor cells to hijack neuronal activity for their own proliferation and invasion. This integration not only disrupts normal neural circuitry but also facilitates tumor progression by exploiting the brain's existing network [2, 14].

Cancer neuroscience [15] investigates the interactions between tumoral processes and brain connectivity to develop better diagnostic, monitoring, therapeutic, and rehabilitative strategies [16, 17]. Structural connectivity (SC) can be determined from diffusion MRI (dMRI) and attempts to identify the physical WM projections between cortical networks [18]. While not without limitations [19], SC can be characterized by the normalized number of streamlines (NOS) of the connections between each pair of cortical regions [20]. Glioblastomas have been shown to disrupt SC throughout the brain, spreading beyond the tumor site and affecting normal tissue, which is associated with worse patient performance and decreased survival rates [9]. Functional connectivity (FC) can complement SC by representing correlated patterns of blood oxygen level–dependent (BOLD) activity across brain regions and provides statistical associations between regional hemodynamic time courses acquired with resting‐state fMRI (rs‐fMRI) [21]. Few studies have explored whole‐brain FC in glioma [22, 23, 24, 25, 26]. Daniel et al. [10] first hypothesized that the strength of FC within glioblastoma is predictive of overall survival. They later highlighted that homotopic FC disruptions in glioma patients are associated with tumor malignancy and overall survival [11].

The complementarity of SC and FC suggests that their joint analysis could provide additional understanding on the mechanisms through which gliomas consistently affect both brain structure and function [27]. However, there is currently a lack of frameworks seemingly allowing for the joint analysis of multiple connectivity modalities. Ideally, such a framework would allow to understand common patterns of changes in SC and FC and detect their alterations at the individual level. Neural network frameworks are emerging as important tools for analyzing multimodal and integrated brain connectivity in pathology. Variational autoencoders (VAEs) [28] have previously been applied to various imaging tasks, including brain tumor segmentation, spatiotemporal feature acquisition, topological connectivity reconstruction, and the generation of realistic and diverse data reflecting true distributions [29, 30, 31]. VAEs can be used to integrate multimodal neuroimaging data into lower dimensional latent spaces, enabling interpretable representations [32, 33, 34]. Furthermore, VAEs can support anomaly detection frameworks [35], which aim to identify deviations from normal brain connectivity patterns. Thus, applying VAE to explore structural–functional brain connectivity in glioma patients may reveal patterns of impairment and provide insight into the relationship between SC, FC, and tumor location.

This study aims to explore the potential of VAEs for detecting joint patterns of change in SC and FC in gliomas. Our primary focus is the integrated anomaly detection of SC, FC, and FC + SC at the single‐subject level and its relationship to tumor and surrounding tissues. We also aim to identify glioma‐related connectivity patterns that could inform personalized care by characterizing configurations of tumor‐induced network perturbations that are consistent across subjects. We expect these patterns to capture both local effects near the tumor and distant changes in structurally or functionally connected regions. For completeness, we include an exploratory analysis of low‐dimensional connectivity representations in the Supporting Information; this material is not used for inference in the main manuscript. By delineating individual connectivity profiles, this approach could be further explored in the future for personalized strategies for diagnosis, monitoring, and treatment planning.

## 2 Material and Methods

In the following sections, we will explain the characteristics of the data used in this study and how these were preprocessed for the subsequent derivation of SC and FC matrices. Afterwards, we will explain how we first trained VAEs to learn joint FC and SC patterns in healthy controls and then applied them to detect anomalies in the tumor patients' after transfer learning. Finally, we will describe how we investigate latent space features that are representative of clinically relevant subgroups of subjects.

### 2.1 Study Cohort

The data of this project was composed of two subject groups. The first dataset comprised newly diagnosed glioma patients acquired at the Neurologic Clinic in Padua University Hospital between July 2017 and April 2021. The second data collection was derived from the Human Connectome Project (HCP) in Aging (HCP‐Aging) [36] project. Data from the HCP‐Aging pool were selected matching the oncological dataset for sex and age range. This healthy subjects' data collection was employed as the healthy data group for neural network training, validation, and testing.

**Dataset1:** Forty‐one patients (59.5 ± 15 years, 23/18 M/F) with newly diagnosed glioma at different positions (lesion hemisphere: 22 left hemispheres, 14 right hemispheres, 5 bilateral) and grades (I–IV) were enrolled in the study between July 2017 and April 2021 at the Neurologic Clinic in Padua University Hospital. Due to the patients' enrollment period, tumors were classified accordingly to the 2016 WHO classification [37]. All procedures performed were in accordance with the ethical standards of the institutional research committee and with the 1964 Helsinki Declaration and its later amendments or comparable ethical standards. All participants provided written and informed consent in accordance with the local Ethic Committee (*Comitato Etico per la Sperimentazione Clinica della Provincia di Padova*) (No. 2771P prot:0065859/12). As reported in Table 1, of the total dataset cohort, 26 patients presented wild‐type glioblastomas, while 5 patients had the IDH1 mutation. Patients eligible for the study must fulfill the following requirements: (1) new diagnosis of glioma (no recurrences), (2) age equal to or older than 18 years old, (3) no diagnosis of other psychiatric or neurological disorders, (4) collection of specific research MRI acquisitions, including pre and post‐contrast T1‐weighted sequences, T2‐weighted, FLAIR, rs‐fMRI, and dMRI, and (5) absence of macroscopic metallic artifacts in MR images. It is significant to mention that only a subset of 31 individuals from the original oncological dataset was examined within the anomaly detection approach, as 10 subjects were employed for VAE transfer learning steps. Table S1 provides the demographic and clinical information of the 31 test set individuals. Table S2 describes the same information for the 10 subjects enrolled in the transfer learning approach.

**TABLE 1:** Demographic summary table with the number of male and female patients, the number of patients with a specific tumor histology, the number of patients grouped by the lesion type classification (WHO 2016 [37] classification), and the number of patients grouped by the lesion hemisphere position (*n* = number, n.a. = not available).

Age (years)	59.5 ± 15
Gender	
Female (*n*)	18
Male (*n*)	23
Tumor histology	
Astrocytoma (*n*)	2
Glioblastoma (*n*)	29
Glioneuronal and neuronal tumors (*n*)	3
Oligodendroglioma (*n*)	1
Primary diffuse large B‐cell lymphoma of the CNS (*n*)	1
Other (*n*)	2
n.a. (*n*)	3
Tumor grade	
Low (*n*)	6
High (*n*)	32
n.a. (*n*)	3
IDH‐1 mutation status	
Wild type (*n*)	26
Mutated (*n*)	5
n.a. (*n*)	10
Tumor site	
Left (*n*)	22
Right (*n*)	14
Bilateral (*n*)	5

MRI and PET imaging sequences were concurrently acquired at the Nuclear Medicine Unit, Department of Medicine, University Hospital of Padua, using a Siemens Biograph mMR (Siemens Medical Solutions USA Inc.) PET/MRI scanner equipped with a 16‐channel head–neck coil. The MRI protocol included a set of anatomical images, diffusion‐weighted images (DWIs), and rs‐fMRI images. Details are described in Supporting Information S1.1.

**Dataset2:** A total of 200 subjects (59.29 ± 14.28 years, 112/88 M/F) were selected from the HCP‐Aging dataset to match Dataset1 sex and age range. Subjects included these images: clinical structural imaging (T1w and T2w), dMRI, rs‐fMRI, and task fMRI. The HCP‐A imaging protocol was acquired with a standard Siemens 3T Prisma scanner with a 32‐channel head coil. HCP‐Aging project enrolled individuals in the age range [36–100] years, according to the second HCP release (Lifespan HCP 2.0 Data Release) instructions and collected healthy brains. All HCP participants gave full written informed consent prior to the data collection, following Washington University‐University of Minnesota (WU‐Minn HCP) Consortium ethical guidelines. All procedures were performed in accordance with relevant guidelines and regulations. All the study protocols have been approved by the local Institutional Review Board (IRB) at Washington University in St. Louis. To be part of the study, participants must not present the following criteria: diagnosis or treatment for major neuropsychiatric or neurological disorders, depression, cognitive impairments, learning disabilities, or severe medical conditions [36]. Further details are provided in Supporting Information S1.2.

### 2.2 MRI Processing

For Dataset1, structural images were linearly aligned to the T1w image through the Advanced Normalization Tools (ANTs) [38] (v. 2.0.1) toolbox. This registration process was necessary for the lesion mask delineation, manually performed by an expert neuroradiologist with more than 5 years of experience. ITK‐SNAP software (http://www.itksnap.org/) supported the neuroradiologist in the procedure. The MR‐visible lesion segmentation was further subdivided into the tumoral core (T) and the edematous tissue (O). The tumor core featured the contrast‐enhancing, non‐contrast‐enhancing, and necrosis areas (when present). This classification enabled statistical analyses accounting for different glioma tissues. Lesion masks were also utilized for preprocessing steps and advanced analyses, to perform a lesion‐aware and subject‐specific registration. Because atlas‐based approaches can be affected by tumor‐induced mass effects, we adopted a tumor‐aware normalization procedure. Lesion masks, including edema, enhancing/nonenhancing core and necrosis, were manually delineated by an expert neuroradiologist using four structural MRI sequences: T1w, post‐contrast T1w, FLAIR and T2w. Each mask was then dilated by 1 mm and used as an exclusion zone during nonlinear registration to MNI152 2009c symmetric [39] and MNI152 symmetric [40] templates using ANTs [38]. Voxels within the dilated mask were excluded from the cost function (mutual information and cross‐correlation), therefore ensuring that tumor‐affected tissue did not influence the estimated deformation fields. Details about structural processing are described in Supporting Information S1.3.

dMRI images of both datasets were processed using the diffusion pipeline detailed in Supporting Information S1.4, aiming to generate subject‐specific tractograms.

rs‐fMRI data of Dataset1 underwent a standard preprocessing as detailed in Supporting Information S1.5. rs‐fMRI data of Dataset2 were already preprocessed and aligned to the MNI152 space [40] according to the minimal processing pipeline [41, 42]. To ensure comparability with the oncological data, additional processing steps were applied to the rs‐fMRI data and are described in Supporting Information S1.6. It has to be mentioned that 45 HPC subjects were later removed after the quality check controls of the processing steps.

### 2.3 SC and FC

The Schaefer [43] cerebral cortex atlas (100 parcels, 17 Yeo Networks [44] per hemisphere) and 10 subcortical regions of the AAL3 atlas [45] (left and right thalamus, caudate, putamen, pallidum, hippocampus) were unified and selected as a global parcellation scheme. Cortical and subcortical regions were employed given the usual tumor localization, spatial patterns, and infiltrative nature [46, 47, 48, 49, 50].

For each patient, brain connectivity was assessed using both SC and FC. The structural connectome matrix was quantified according to the NOS metric (SC), normalized by the total number of tracts of the connectome (i.e., 10 million). The process involved the overlay of the atlas‐based parcellation onto the individual whole‐brain tractogram and the assessment of the NOS connection strength. The result was a 210 × 210 SC matrix. It is worth mentioning that SC matrices entries counting one streamline were set to zero. FC was estimated as the temporal similarity between dynamic neural activity patterns of brain regions, using Pearson correlation. The procedure entailed the overlay of the Schaefer‐AAL3 parcellation onto the rs‐fMRI preprocessed volume of each subject in both pools. In this case, FC referred to the statistical relationship between rs‐fMRI physiological time signals of couples of parcels. Pearson correlation was computed between each mean time series of pair regions of interest (ROIs), resulting in a 210 × 210 matrix. For oncological patients, voxels in overlap with the necrotic areas were discarded from the Pearson correlation calculation, whereas, given the shorter period of acquisition for each HCP rs‐fMRI run compared with the oncological, the final FC matrix for each HCP subject entailed the mean between the FC matrices of the AP and PA first runs.

For oncological functional data (Dataset1), Schaefer and AAL3 atlases were aligned to the symmetric MNI152 2009c atlas [39]. The Schaefer and AAL3 atlases were also directly coregistered to the symmetric MNI152 2009c atlas employing a transformation matrix previously derived from the registration of the MNI152 symmetric atlas and the symmetric MNI152 2009c atlas (Rigid/MI → Affine/MI → SyN/CC in ANTs [38]). Regarding oncological diffusion images (Dataset1), Schaefer and AAL3 parcellations were aligned to the single subject B0 volume, applying a single interpolation step using previously derived transformation matrices. This transform included the registration from the MNI152 symmetric atlas to the individual T1w image, then to the individual T2w image, and finally an affine transform between the T2w and the B0 images.

For HCP‐Aging subjects (Dataset2), Schaefer and AAL3 parcellations were aligned to the individual T1w using FSL software [51], with transformations previously estimated by the HCP minimal processing pipeline [41].

### 2.4 Data Normalization

The variational autoencoder approach entailed the concatenation of the upper triangular FC and SC connectivity matrices for each subject. SC was transformed following Civier and colleagues' method [52] to make its distribution more similar to that of FC. This was performed by applying a data‐driven transformation based on a power‐law model. This transformation proved essential to provide SC and FC simultaneously as input to the VAE [52].

As VAEs typically require normalized data as input, we compared min‐max, *z* score, and inverse hyperbolic tangent normalization. The latter provided the best results and was used throughout this work.

### 2.5 Variational Autoencoder

#### 2.5.1 Theory

VAE is an unsupervised deep learning framework trained to learn complex data representation [53]. A VAE architecture comprises an encoder and a decoder. The encoder represents the input with latent variables in a low‐dimensional space, hence only learning key features that represent the input. The decoder reconstructs an output based on a sampling of the latent variables. The training aims to minimize a loss term, composed of a reconstruction loss and the Kullback–Leibler (KL) divergence between the latent variables' distributions and independent normal distributions [28]. An interesting use of VAEs is anomaly detection: When a VAE is provided with previously unseen input (i.e., an anomaly), it likely removes it in its reconstruction, leading to a high dissimilarity with the input. As such, anomalies can be detected by monitoring the reconstruction error. The latent space of a VAE contains the core features learnt to represent the input data. As such, we aimed to explore the latent space to fingerprint different connectivity profiles. β VAE was introduced instead of VAE to learn disentangled representations. β VAE introduces a hyperparameter β that controls the trade‐off between the reconstruction term and the KL divergence term in the VAE objective function [54]. It is useful to normalize β by latent space size (*M*) and input size (*N*), to compare its different values across different latent layer sizes and different datasets; thus, $\beta_{\text{NORM}}=\beta \frac{M}{N}$. $\beta \frac{M}{N}$ VAE architecture with *β* = 1 was selected for the study, thus a VAE frame with a normalization coefficient including latent size and input size.

#### 2.5.2 Architecture

**FIGURE 1:**
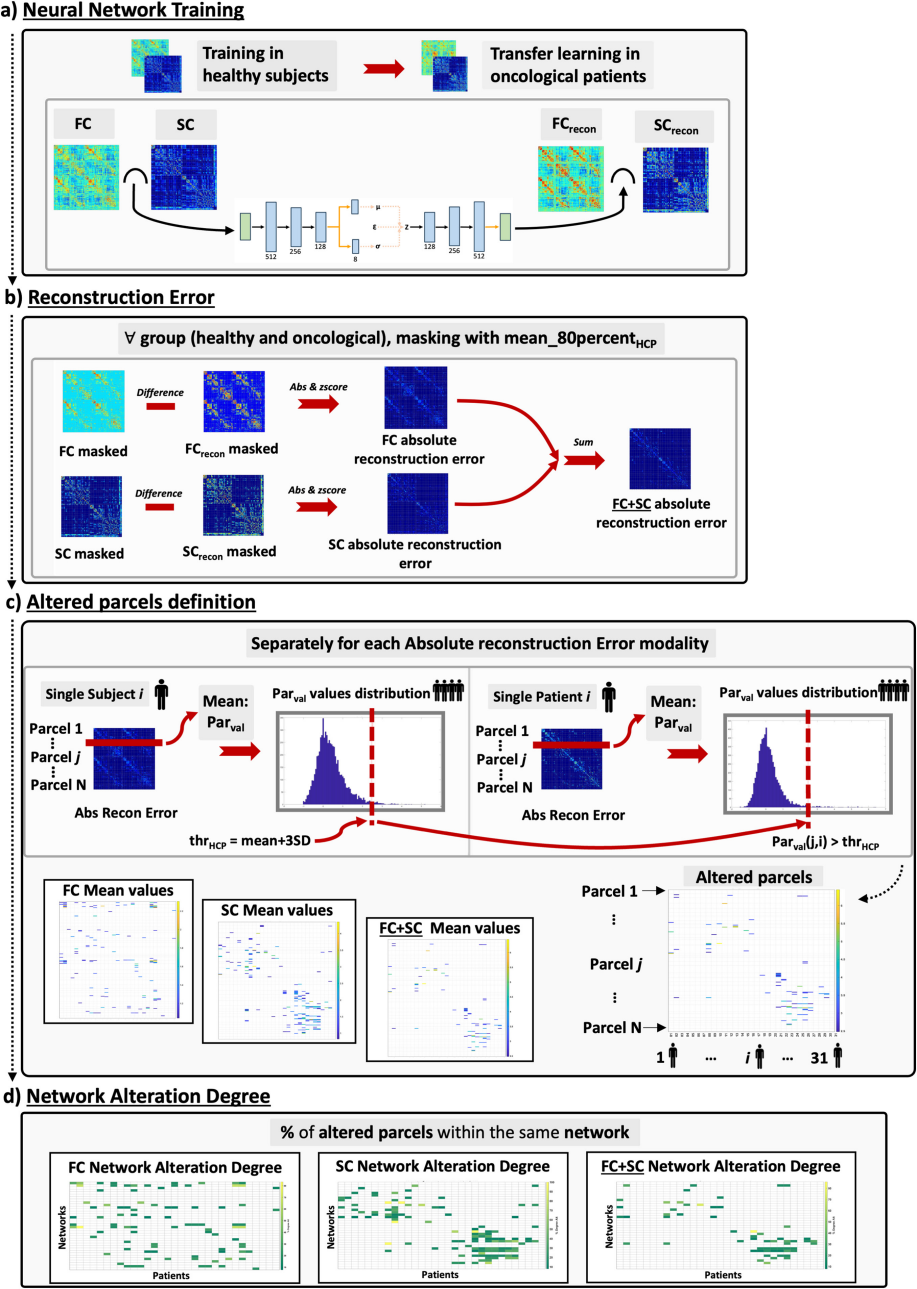
Workflow illustration of the analysis procedure. (a) Variational Autoencoder framework applied to HCP and Oncological data. (b) Approach for deriving the reconstruction error measure for each connectivity mode and each patient (after ad hoc normalization). (c) Anomaly detection procedure for defining altered connectivity parcels (i.e., each matrix row). At first, for each connectivity mode, a threshold is derived from HCP healthy data. Then, oncological anomaly parcels are set according to the defined healthy threshold. (d) Network Alteration Degree, representing the percentage of altered parcels within the same network among the patients, for each connectivity mode.

Regarding the VAE application on SC‐FC interplay, the neural network was designed with eight latent variables, and the encoder and decoder were both composed of linear layers (Figure S1). Each linear layer included a Leaky Rectified Linear Unit (Leaky ReLU) activation function. Leaky ReLU is an activation function where the negative section allows a small gradient instead of being completely zero. The number of nodes of the encoder was equal to 512, 256, and 128. Comparably, the decoder was composed of layers of 128, 256, and 512 nodes. The latent space dimension was defined as equal to eight nodes. The model was trained on 87 subjects of the HCP dataset, with every training input equal to the concatenation of the normalized zFC and zSC upper triangular matrices (described in Data Normalization section). A batch size equivalent to 8 was selected to divide the training set. Number of epochs was set equal to 2000, and patience equal to 750. Adam optimizer with a learning rate (lr) equivalent to $1e^{-3}$ was applied [55]. VAE performances were evaluated minimizing mean squared error loss. The model was implemented in PyTorch (http://pytorch.org/). Figure S1 provides a graphical representation of the VAE just described. The model was then validated on 37 HCP subjects and finally tested on 31 HCP subjects. Therefore, for each subject of the test set, a reconstructed FC and SC connectivity matrix was derived (FC_recon_ and SC_recon_). SC_recon_ presented some values slightly below zero and were therefore set to 0. It is worth mentioning that the model provided the inverse hyperbolic tangent version of FC and SC. For this reason, final reconstructed matrices were derived after the employment of a hyperbolic tangent. It is also important to note that SC reconstructed was the transformed version of the SC matrix, due to the initial application of the Civier procedure [52]. The right side of panel 1 of Figure 1 abstractly represents the approach previously described. Details of the reconstruction quality assessment procedures and different VAE architectures are provided in Supporting Information S1.7.

### 2.6 Oncological Transfer Learning

With the transfer learning approach, a neural network trained on a certain dataset (i.e., healthy subjects) can be adapted to a new domain (i.e., patients). The neural network is usually fine‐tuned using a small training set from the target domain [56]. In our study, training was performed in Dataset2 and then fine‐tuned on Dataset1 using selected glioma patients with limited abnormalities. These glioma subjects were chosen based on the results of a different study, which investigated SC and FC alterations in this cohort with a different approach [57].

Figure 1a summarizes the steps of transfer learning on oncological patients, which are also reported in detail in Supporting Information S1.8. In summary, within the 10 training glioma subjects, transfer learning was performed with seven selected glioma patients. A later validation on three selected brain tumor patients was performed [58]. The final test on the remaining 31 subjects was performed to obtain the reconstructed FC_recon_ and SC_recon_ connectivity matrices. It is also important to note that SC_recon_ was the transformed version of the SC matrix, due to the initial application of the Civier procedure [52].

### 2.7 Anomaly Detection

We performed anomaly detection by comparing the matrices reconstructed from the VAE to the original connectivity matrices and determining which edges exceeded a predefined distance threshold based on healthy subjects.

The first step to perform anomaly detection was to establish the normal distribution of brain connections. To this end, group‐average FC and SC matrices were quantified from HCP test data, creating the meanFC_HCP_ and meanSC_HCP_ matrices. The group‐average matrices were later thresholded, retaining only connections associated with FC and SC weights over the 80th percentile of the meanFC_HCP_ and meanSC_HCP_ weights distribution [59], deriving meanFC_80percent_HCP_ and meanSC_80percent_HCP_. FC cut‐off choice was fixed according to van den Heuvel et al. [60]. Consistently, the 80th percentile threshold value was set for meanSC_HCP_. As highlighted in de Brito Robalo et al. [61], it is expected that applying thresholding leads to more consistent network architectures by reducing false positives and improving precision in network analysis.

From test HCP data, FC and SC connectivity matrices of each subject were reconstructed as already described. Thus, for both connectivity modalities, the absolute differences between the original FC/SC and its reconstructed FC_recon_/SC_recon_ were computed, obtaining FC and SC difference matrices, and masked with the meanFC_80percent_HCP_ and meanSC_80percent_HCP_ matrices. Then, single modality difference matrices underwent ad‐hoc normalization to ensure reliable statistics. Integrated connectivity measure was derived by summing the FC and SC difference matrices and creating the FC + SC difference matrix. Oncological test patients' FC and SC matrices underwent a similar procedure. The process is illustrated in Figure 1b and described in Supporting Information S1.9.

To identify abnormalities in each modality (FC, SC, FC + SC) of the oncological dataset, modality‐specific thresholds (thr_HCP_) were defined as the mean value plus three standard deviations (mean + 3SD) of the difference between the original parcels‐derived connectivity matrix and its reconstruction in the HCP test dataset. It should be noted that thresholding of difference matrices was applied in order to identify connections that deviate from expected behavior. For further details, refer to Supporting Information S1.9.

Eventually, for each connectivity modality, a vector (i.e., the vector of the impaired parcels) containing all the parcels that were found to be potentially altered according to the thr_HCP_ was created. Vectors corresponding to the analyzed patient were arranged side by side to create a unified matrix with dimensions of 210 × 31.

All statistics were performed with in‐house MATLAB scripts (MATLAB 2024a, The MathWorks Inc., Natick, MA, USA) and in‐house Python modules (Python Software Foundation. Python Language Reference, version 3.11.5. Available at http://www.python.org).

### 2.8 Degree of Alteration of Each Network

Once the connectivity altered parcels were obtained, a measure of overall alteration for each Yeo [44] and Subcortical network (i.e., node) was derived. The global number of networks was equal to 18 for each hemisphere. It is worth highlighting that, for the sake of simplicity, subcortical regions were merged into two separate networks (left and right hemispheres). Concerning the graph analysis theory of node degree [1], for each network, the degree of alteration was evaluated as the percentage of altered parcels within the same network. This value was indicated as network alteration degree (NAD). Supposing each network *n* composed of *K* parcels, a measure of NAD was defined as follows:

(1) $${\mathrm{NAD}}_{n}=\left(\frac{1}{K}\sum_{k=1}^{K}{\text{Parcel}}_{\text{impaired}}\left(k\right)\right)\times 100$$

With this computation, the measure of alteration degree represents, for each network (i.e., node), the amount of alteration degree given by the parcels that belong to the same network.

The computation of Equation (1) enabled linking the severity of the state of structural–functional (FC + SC) abnormalities within each network to their stand‐alone connectivity (i.e., FC and SC) alterations. This allowed us to explore glioma abnormalities in FC and SC and in their integration FC + SC, because the single connectivity interconnections are intricate [62].

In Figure 1d, the general strategy to define the altered parcels for every connectivity modality is displayed. NAD was calculated for each test patient for each connectivity modality (FC, SC, and FC + SC). Regions' alterations were additionally grouped according to the tissue type (tumor, edema, or normal appearing) in overlap with them. Featuring lesional tissues in overlap with networks offered a better conception of the glioma's behavior because of its penetrative spread [8]. It is essential to underline that each patient was provided with a lesion mask distinguishing two main lesion tissue types, thus tumoral core (T) and edematose (O) tissue.

Furthermore, a measure of patient global disruption (GD) could be assessed by deriving the number of altered networks within each subject. Given *N* networks, for each subject *j*, the GD was computed as follows:

(2) $${\mathrm{GD}}_{j}=\sum_{n=1}^{N}\left({\mathrm{NAD}}_{n}>0\right)$$

Referring to FC, SC, and FC + SC connectivities, three GD indexes were obtained for each patient (GD_FC_, GD_SC_, and GD_FC+SC_).

Given the general aim to investigate the relation between single and integrated connectivity, a final metric of overlap index (OI) was computed to quantify, for each subject j, the percentage of networks concurrently abnormal for FC and FC + SC (FC ∩ FC + SC) compared with the GD derived from FC (GD_FC_). The same overlap index was derived for SC, to evaluate the variation of SC and FC + SC alterations overlap (SC ∩ FC + SC) and GD derived from SC (GD_SC_). General equations are shown below:

(3) $${\mathrm{OI}}_{{\mathrm{FC}}_{j}}=\frac{{\mathrm{GD}}_{\mathrm{FC}\cap {\underline{\mathrm{FC}+\mathrm{SC}}}_{j}}}{{\mathrm{GD}}_{{\mathrm{FC}}_{j}}}\cdot 100$$

(4) $${\mathrm{OI}}_{{\mathrm{SC}}_{j}}=\frac{{\mathrm{GD}}_{\mathrm{SC}\cap {\underline{\mathrm{FC}+\mathrm{SC}}}_{j}}}{{\mathrm{GD}}_{{\mathrm{SC}}_{j}}}\cdot 100$$

Pearson correlation was used to test the correlations between GD indices and tumoral (T) volume and lesion (T + O) volume. Similarly, Spearman correlation analysis was performed to measure the degree of association between OI values and tumoral (T) and lesion (T + O) volume. All correlation results underwent multiple comparison corrections. Statistically significant p‐value was set equal to a=0.05.

To check on the utility of the transfer learning approach, the trained model on HCP data was also directly applied to glioma patients. Details concerning the fine‐tuning impact are outlined in Supporting Information S1.10.

An exploratory analysis of low‐dimensional connectivity representations to identify patients' subgroups is provided in the Supporting Information S1.11. Given their exploratory nature, these analyses are not used for inference (i.e., to draw conclusions) in the main manuscript.

## 3 Results

**FIGURE 2:**
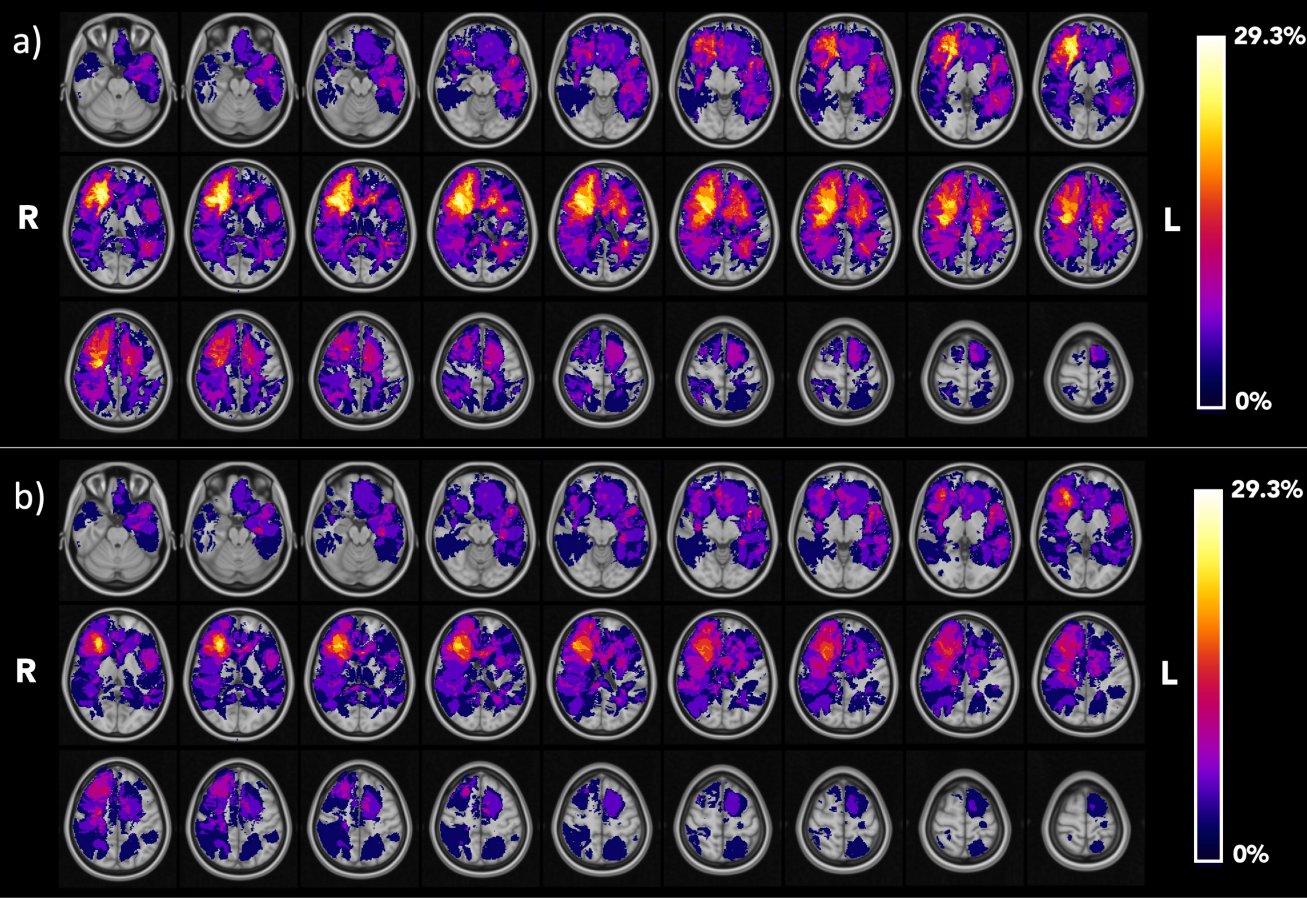
Lesion frequency map across patients' cohort. (a) Frequency map of tumor lesions including edema area (T + O); (b) map of tumor core, excluding edematose tissues (T). Maps are overimposed to the MNI atlas (gray scale). Radiological convention.

Figure 2 shows the frequency maps of the lesions/tumors in the population of 31 test patients. More frequently impacted regions predominantly involved association regions with high FC, such as the left temporal, the right temporal, and the right frontal lobes. The maximum lesion (T + O) overlap value among patients was 29.3%, while the highest tumor (T) overlay value was equal to 26.1%. These results aligned with the expected frequency distribution reported by Mandal et al. [46, 49].

### 3.1 Reconstructing HCP Connectivity With VAE

The FC and SC matrices of each subject were concatenated and supplied as inputs to the VAE. The VAE provided as output a reconstruction of the same matrices based on their low‐dimensional latent space representation. Figure S2 compares the mean FC and mean SC of the HCP subjects to the corresponding FC_recon_ and SC_recon_. By visual inspection, reconstructed edges along the main and superior diagonals are characterized by a lower amplitude compared with the original values. Edges off the diagonal show comparable structures and amplitudes. Spearman correlations were computed between meanFC and meanFC_recon_ and meanSC and meanSC_recon_, with values of 0.927 (*p* value < 0.05) and 0.929 (*p* value < 0.05), respectively. In the Supporting Information, we additionally evaluate the performance of the VAE when separately training it on SC (Figure S3a) or FC (Figure S3b). In this case, the Spearman correlation values were lower, both equal to 0.912 (*p* value < 0.05). Reconstruction quality assessment results were also examined and outlined in Supporting Information S2.1 and Figure S4.

### 3.2 Anomaly Detection on Oncological Data

#### 3.2.1 Global Connectivity Impairments

The anomaly detection procedure developed for the glioma dataset provided a measure of deviation from a reference group (i.e., healthy subjects) for each connectivity modality. The first result, shown in Supporting Information S2.2, demonstrates a linear association between GD and OI indices with tumor/lesion volumes.

**FIGURE 3:**
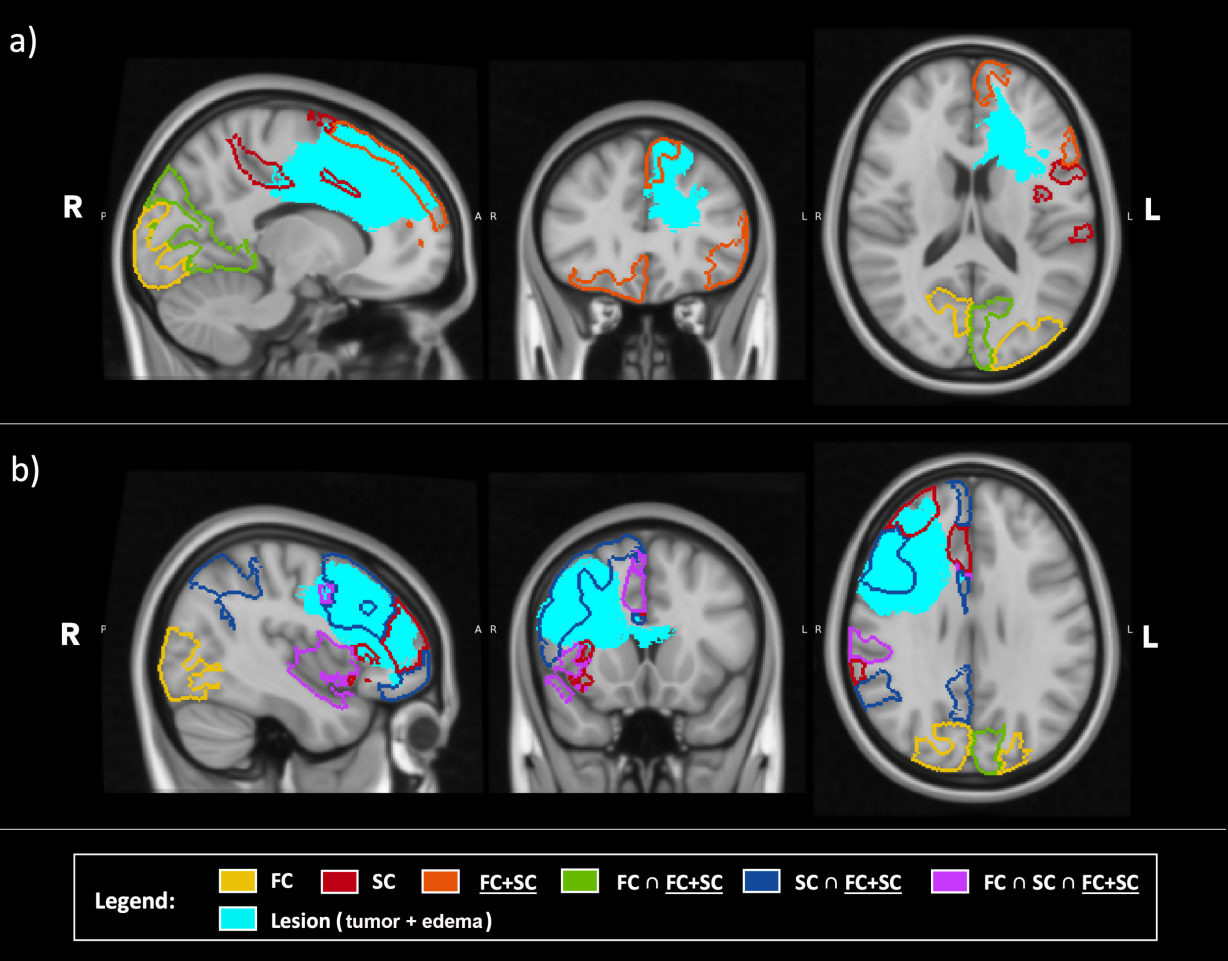
Abnormal networks for two representative patients. (a) Overlap between lesion location and altered networks for a patient with a left hemisphere glioma (Patient ID 2, Table S1). (b) Overlap between lesion location and altered networks for a patient with a right hemisphere glioma (Patient ID 25, Table S1). For both panels, yellow are networks altered by FC only, red are networks altered by SC only, orange are networks altered by FC + SC only, green are networks altered by FC and FC + SC (FC ∩ FC + SC), blue are networks altered by SC and FC + SC (SC ∩ FC + SC), and purple are networks altered by FC, SC, and FC + SC (FC ∩ SC ∩ FC + SC). The maps are superimposed on the MNI atlas (gray scale). Radiological convention. In the first patient, who has a glioma in the left hemisphere, Figure 3a shows the lesion (light blue) and the altered networks according to FC, SC, FC + SC, and their overlap. This patient does not present altered networks in overlap with the tumor. Within edema, Left Salience/Ventral Attention‐A is abnormal according to SC, Left Default‐A presents FC + SC alteration. In healthy tissues, Visual Central, Visual Peripheral and Limbic networks are mostly affected. Specifically, Left Visual Central is altered for FC, Left Visual Peripheral is impacted for both FC and FC + SC, Right Visual Peripheral is altered according to FC and Right Limbic‐B is affected for FC + SC. For the second patient, who has a glioma in the right hemisphere, Figure 3b demonstrates the same network configurations. Within the tumor, right networks are affected. Right Salience/Ventral Attention‐A is affected according to FC, SC, and FC + SC. Right Salience/Ventral Attention‐B is affected according to SC. Right Control‐A, Control‐B and Default‐A are impacted by SC and FC + SC. The patient does not present abnormalities within edema. Outside of the lesion, Left and Right Visual Central and Right Visual Peripheral are impacted by FC, while Left Visual Peripheral is impaired according to FC and FC + SC.

Figure 3 illustrates the altered networks and their direct connections to the lesion for two representative patients. Results highlight the variability in network alterations across different patients and lesion locations, emphasizing the importance of individualized assessments in clinical settings.

The distribution of network alterations across all patients in the test set is shown in Figure S5, where patients are grouped by lesion hemisphere to highlight anomalies and patterns. Altered networks are categorized into groups overlapping with the tumor, edema, or healthy tissues. Within the tumor (Figure S5a), it is important to note that patients mostly exhibited concurrent occurrences of alterations in any of the combinations. The Right Control B network showed the highest frequency of coinciding abnormalities in FC, SC, and FC + SC, accounting for 9.7% of patients, and was associated with areas of higher tumor occupancy (Figure S6). Left Control A, Left Default A, and Right Default B showed a frequency of 6.45% for concurrent FC, SC, and FC + SC anomalies. In the edema (Figure S5b), no patients presented simultaneous alterations across all metrics. Single SC network abnormalities were more frequent, with Right Default A showing the highest frequency (12.9%) of isolated SC anomalies. Right Control A and Right Control B regions exhibited significant overlap of SC and FC + SC anomalies. In healthy tissues (Figure S5c), most alterations were assigned to FC alone, particularly in the Left and Right Central Visual and Peripheral Visual networks, which were frequently affected across subjects but minimally altered in tumor and edema regions (Figure S5a,b). No subjects exhibited concurrent alterations in FC, SC, and FC + SC within these healthy tissue networks.

#### 3.2.2 Investigating the Relationships of Single and Integrated Connectivity

**FIGURE 4:**
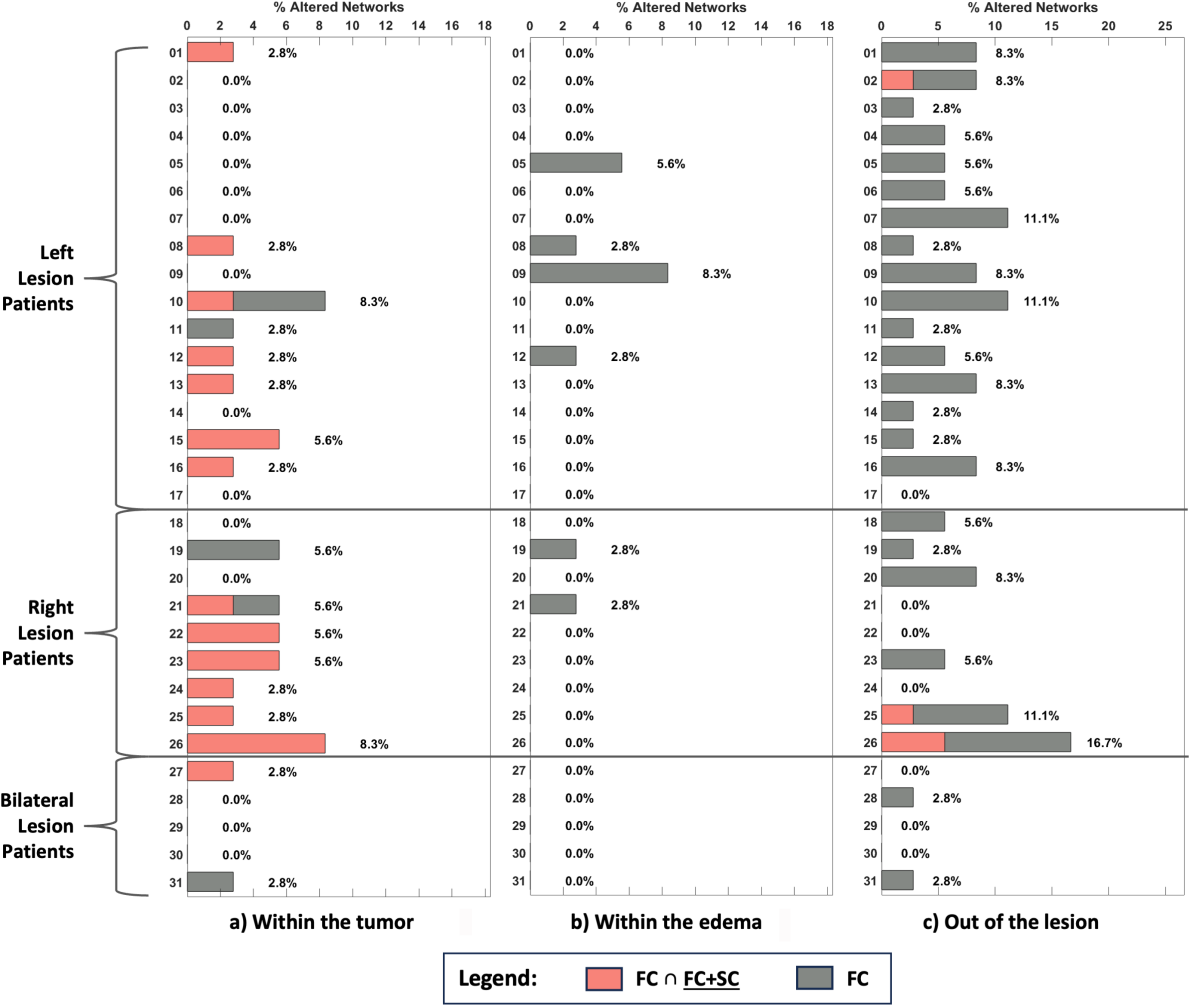
(a) Bar plot of the percentage of FC ∩ FC + SC and FC altered networks within the same patients, for networks overlapping with the tumor (T). (b) Bar plot of the percentage of FC ∩ FC + SC and FC altered networks within the same patients, for networks overlapping with the edema (O). (c) Bar plot of the percentage of FC ∩ FC + SC and FC altered networks within the same patients, for networks overlapping with healthy tissues. Patients are grouped according to lesion hemisphere position.

For each patient, Figure 4 illustrates the proportion of networks with both FC and FC + SC alterations (denoted as FC ∩ FC + SC) and those with only FC impairment. The affected networks are separated based on their overlap with the tumor core (T) (Figure 4a), edema (O) (Figure 4b), or healthy tissues (Figure 4c). In networks overlapping the tumor (Figure 4a), most patients exhibited alterations in FC ∩ FC + SC. Conversely, no subjects had FC ∩ FC + SC altered networks overlapping with edema (Figure 4b). For networks outside pathological areas (Figure 4c), most patients exhibited only FC abnormalities, particularly those with left hemisphere lesions. Overall, networks within healthy tissues tended to show more FC impairments (Figure 4c), whereas networks within the lesion were less affected in terms of FC (Figure 4a,b).

**FIGURE 5:**
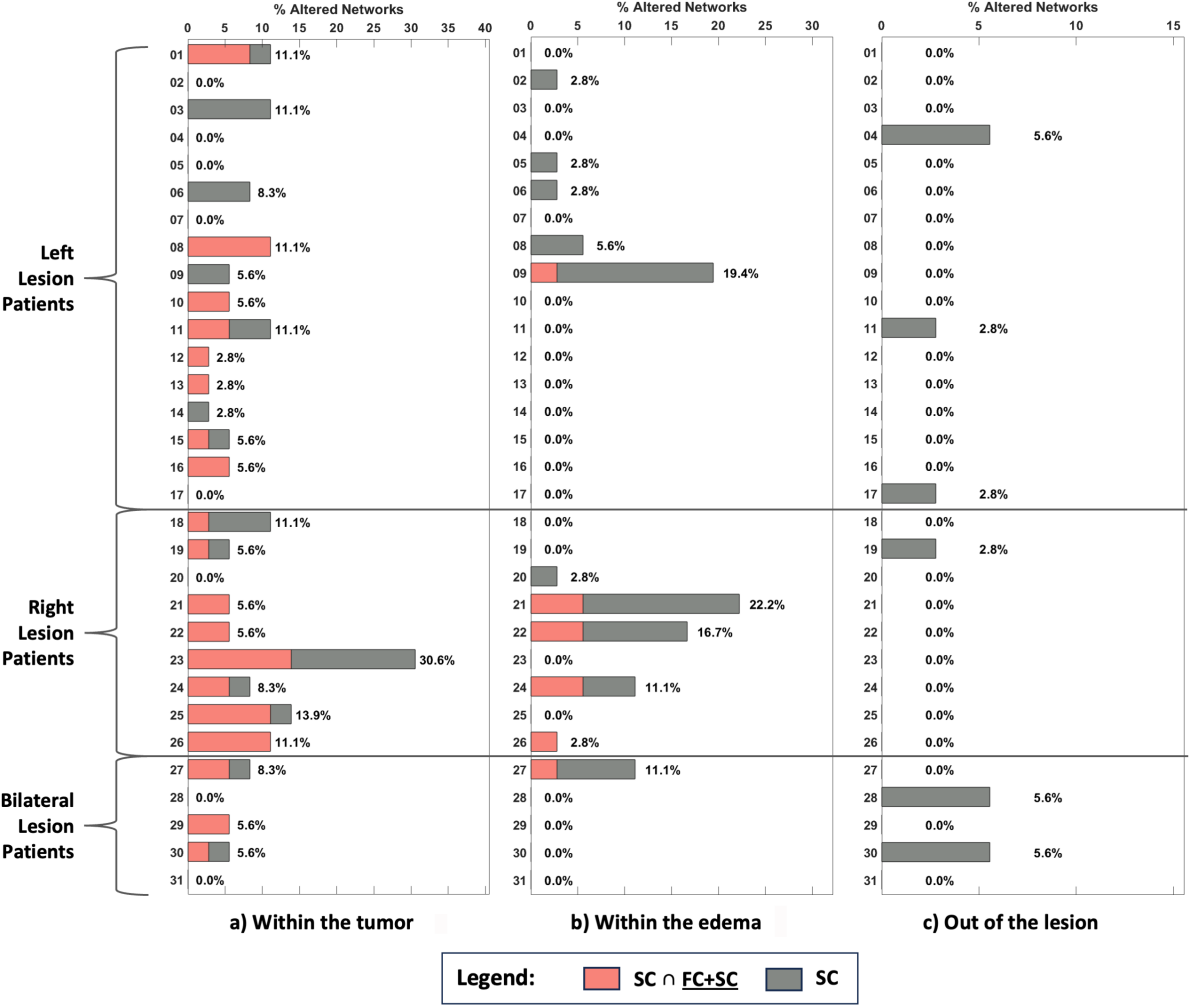
(a) Bar plot of the percentage of SC ∩ FC + SC and SC altered networks within the same patients, for networks overlapping with the tumor (T). (b) Bar plot of the percentage of SC ∩ FC + SC and SC altered networks within the same patients, for networks overlapping with the edema (O). (c) Bar plot of the percentage of SC ∩ FC + SC and SC altered networks within the same patients, for networks overlapping with healthy tissues. Patients are grouped according to lesion hemisphere position.

Figure 5 explores the relationship between anomalies detected with SC only and combined FC + SC connectivity. Figure 5a shows that, within the tumor, most patients exhibited SC damages that partially overlapped with FC + SC alterations (denoted as SC ∩ FC + SC). Patients with right hemisphere lesions showed more significant SC impairments (10.2% of brain regions on average) compared with those with left hemisphere lesions (4.9% of brain regions on average). In regions in contact with edema (Figure 5b), patients with right hemisphere lesions again displayed more network alterations, with both SC alone and SC ∩ FC + SC damages. Outside the lesion (Figure 5c), SC changes were present without corresponding FC + SC alterations.

Comparing Figures 4 and 5, it is evident that within the lesion, SC is typically more impaired than FC (Panels a and b of each figure). Instead, FC is more likely to be affected out of the lesioned tissues (Panel c of each figure). Moreover, FC is generally much more consistent with FC + SC abnormalities. Additionally, networks in contact with edema do not present FC ∩ FC + SC damages, while areas out of the lesion do not show SC ∩ FC + SC affections. Overall, there is less agreement between anomalies detected with SC alone and with combined connectivity, as compared with those detected with FC alone.

**FIGURE 6:**
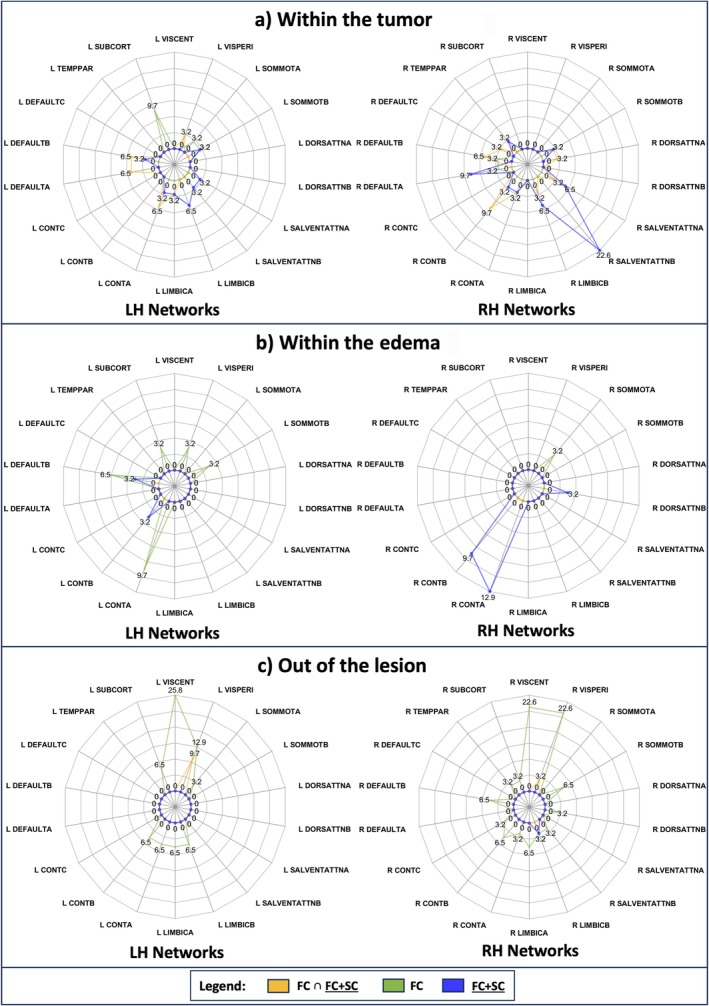
In general, the spider plot represents the percentage of patients with altered connectivity associated to each Cortical and Subcortical Network. Networks are grouped according to left and right hemisphere (left and right side of the image). For each pair of spider plots displayed in the same row, values range is the same. As illustrated in the legend, FC ∩ FC + SC in yellow, FC in green, and FC + SC in blue. (a) The percentage of patients with altered network overlapping with the tumor (T) is depicted. (b) The percentage of patients with altered network in overlap with the edema (O) is displayed. (c) The percentage of patients with altered network out of the lesion. VisCent = Visual Central network; VisPeri = Visual Peripheral network; SomMotA = Somatomotor‐A network; SomMotB = Somatomotor‐B network; DorsAttnA = Dorsal Attention‐A network; DorsAttnB = Dorsal Attention‐B network; SalVentAttnA = Salience/Ventral Attention‐A network; SalVentAttB = Salience/Ventral Attention‐B network; LimbicB = Limbic‐B network; LimbicA = Limbic‐A network; ControlA = Control‐A network; ControlB = Control‐B network; ControlC = Control‐C network; DefaultA = Default‐A network; DefaultB = Default‐B network; DefaultC = Default‐C network; TempPar = Temporal Parietal network; Subcort = Subcortical network.

**FIGURE 7:**
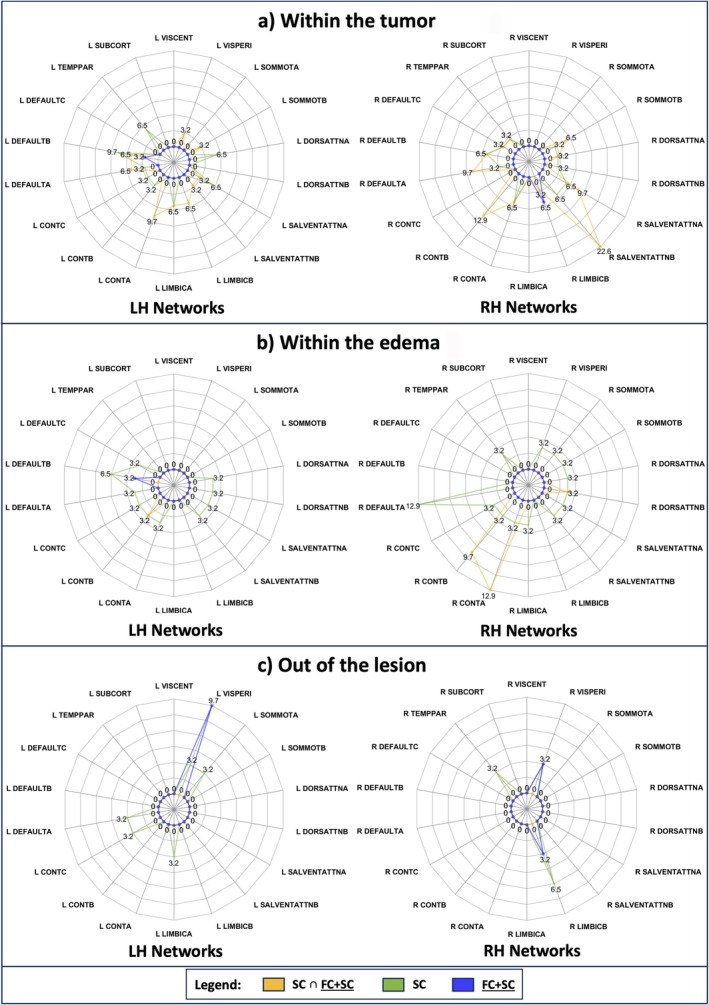
In general, the spider plot represents the percentage of patients with altered connectivity associated to each Cortical and Subcortical Network. Networks are grouped according to left and right hemisphere (left and right sides of the image). For each pair of spider plots displayed in the same row, values range is the same. As illustrated in the legend, SC ∩ FC + SC in yellow, SC in green, and FC + SC in blue. (a) The percentage of patients with altered network overlapping with the tumor (T) is depicted. (b) The percentage of patients with altered network in overlap with the edema (O) is displayed. (c) The percentage of patients with altered network out of the lesion. VisCent = Visual Central network; VisPeri = Visual Peripheral network; SomMotA = Somatomotor‐A network; SomMotB = Somatomotor‐B network; DorsAttnA = Dorsal Attention‐A network; DorsAttnB = Dorsal Attention‐B network; SalVentAttnA = Salience/Ventral Attention‐A network; SalVentAttB = Salience/Ventral Attention‐B network; LimbicB = Limbic‐B network; LimbicA = Limbic‐A network; ControlA = Control‐A network; ControlB = Control‐B network; ControlC = Control‐C network; DefaultA = Default‐A network; DefaultB = Default‐B network; DefaultC = Default‐C network; TempPar = Temporal Parietal network; Subcort = Subcortical network.

Analyzing a broader exchange between single and integrated modality anomalies from a network perspective, Figures 6 and 7 display FC, FC ∩ FC + SC, FC + SC, and SC, SC ∩ FC + SC changes within the tumor (Panel a), edema (Panel b), and outside pathological tissues (Panel c). The spider plot in Figure 6 reveals the proportion of patients with FC, FC + SC, and FC ∩ FC + SC damages. In Panel a of Figure 6, Right Control B is featured by simultaneously FC and FC + SC damage (9.7%). Right Default B is still one of the most altered (9.7%), but it also shows independent FC and FC + SC impairments. Left Subcortical networks were altered exclusively according to FC (9.7%). Isolated FC + SC abnormalities not overlapping FC impairments were found in Left and Right Salience/Ventral Attention A, Salience/Ventral Attention B, Limbic B, and Right Default A. In Panel b, Right Control A and Right Control B predominantly showed FC + SC–only anomalies, with no FC and FC + SC overlap. Outside the lesion (Panel c), higher isolated FC impairments were found in Left Central Visual (25.8%) and Right Central Visual (22.6%).

Figure 7 illustrates the percentage of patients characterized by SC, FC + SC, and SC ∩ FC + SC damages. Right hemisphere regions were slightly more damaged both within the tumor and within edema (Panels a and b). SC ∩ FC + SC alterations were predominant within the tumor in Right Salience/Ventral Attention B (22.6%), Right Salience/Ventral Attention A (9.7%), Right Control B (12.9%), Right Default A (9.7%), and Left Control A (9.7%). Panel a also shows that many brain regions exhibited SC impairments extending beyond the SC ∩ FC + SC anomalies. In Panel b, within the edema, most abnormalities are SC stand‐alone. Right Control A and Right Control B are the most SC ∩ FC + SC impaired (accounting for 9.7% and 12.9%). Right Default A is the network with the highest SC‐alone changes (12.9%). Without the lesion, networks unveiled a similar behavior between the left and right hemispheres (Panel c), with absent SC ∩ FC + SC occurrences. In general, SC affects many more networks within the tumor (Panel a) rather than networks outside the lesioned tissues (Panel c). Left and Right Peripheral Visual present high FC + SC damage. Left Limbic A and Right Limbic B show significant SC alteration.

Generally comparing Figures 6 and 7, the combined FC + SC modality is more sensitive to anomalies within the lesioned area, whereas the individual modalities (FC and SC) tend to highlight differences in disparate regions outside the lesion. This lack of consistency between them explains why these differences are not captured in FC + SC.

The effectiveness of the transfer learning approach was evaluated by directly applying the trained model to glioma patients using HCP data. The results of transfer learning are discussed in Supporting Information S2.3 and Figures S7 and S8.

An exploratory characterization of the latent representations is provided in Supporting Information S2.4 and Figures S9–S12.

## 4 Discussion

This study employed a multimodal VAE approach to detect anomalies in whole‐brain functional–structural connectomes. Our approach confirmed the presence of altered networks in regions characterized by a higher lesion frequency, also resulting in areas far away from the lesion site. In particular, the anomaly detection project enriched the study with details regarding alterations in FC, SC, and their combination (FC + SC) with a novel measure of structure–function integration. Our key findings are as follows: (1) FC is more sensitive to glioma‐induced alterations further away from the pathological tissues, while SC is more impaired in the vicinity of tumoral regions, (2) the alterations identified by FC are generally more in agreement with the alterations identified by FC + SC compared with those highlighted by SC. (3) SC abnormalities never overlap with FC + SC out of the tumor, FC and SC separated impairments are partially concurrent within the tumor core, and never occur in other tissues; (4) VAE feature analysis highlights the glioma fingerprint measures associated with more informative networks that do not coincide with the informative networks of healthy controls.

### 4.1 VAE‐Based Reconstruction of HCP Connectivity

The selected VAE parameters setting provided the best MSE train loss corresponding to 0.018 and the best MSE validation loss equivalent to 0.0188. Loss values indicated good model performance. The higher correlation values, proving the improved performance of the neural network when trained with the concatenation of both FC and SC matrices compared with training with either modality alone, can be attributed to the complementary information provided by these two types of data [63, 64]. FC captures patterns of synchronized BOLD activity [21], while SC represents the physical WM pathways that facilitate these interactions [18]. Combining them enhances VAE by integrating stable anatomical backbones (SC) with dynamic functional relationships (FC), reducing spurious FC signals and revealing more meaningful joint patterns. Moreover, the concatenated input increases feature dimensionality, enabling the model to learn more complex, nonlinear relationships, particularly important given the higher intersubject variability of FC. Rather than introducing noise, this multimodal fusion enhances robustness and supports more expressive feature learning across modalities. Finally, improved reconstruction performances with multimodal variational autoencoders were already obtained at voxel level [32, 33, 34]. In fact, our research findings support the idea that nonlinearities between neuroimaging data can be leveraged using deep learning frameworks [65], as the correspondence between different imaging modalities has been shown to be not linear [66].

### 4.2 Detecting Modality‐Specific and Integrated Anomalies in Oncological Data

The anomaly detection procedure was defined to develop a method able to achieve insights into changes in SC and FC, overcoming separate findings derived from FC and SC. Functional and structural information are known to be sparsely linked, with structural properties poorly predicting functional attributes [63, 67]. Helping to understand the functional, structural, and integrated connections within hierarchical network schemes can reveal the complex interplay between the tumor and the brain to offer a better comprehension of the pathophysiology [68, 69].

Networks more frequently affected by a simultaneous change of FC, SC, and FC + SC were mainly associated with a higher lesion frequency in prefrontal cortex regions (Right Control B, Right Default B, Left Control A, Left Default A). It is known that the prefrontal cortex plays a key role in cognitive control functions, which are high‐level cognitive processes serving for working memory/updating, set shifting, and inhibition [70]. It should be highlighted that executive function degradation commonly occurs in brain pathologies predominantly damaging WM [71, 72]. Then, correlation evidence between glioma WM tract damage and executive function affection has already been demonstrated [73]. On the other side, Default Mode Network (DMN)–decreased activities were observed by many groups [74, 75, 76, 77]. Jutten et al. [78] and Meyer‐Baese et al. [79] already addressed the structural–functional glioma impairments in Default region. Nevertheless, their approach focused on individual modalities and not on their integration, and missing a broad connectome perspective. It is also important to notice that Control and Default regions belong to transmodal networks, demonstrated to be sites of stronger structural–functional coupling [80]. This fact could justify the higher simultaneous changes of FC, SC, and FC + SC observed in these areas.

In terms of widespread connectivity impairments, previous studies of SC [9] and FC [11, 26] have separately suggested widespread damage to structural and functional properties, including neurovascular remodeling [81]. However, these studies could not investigate the relationship between individual and integrated connectivity. The SC and FC affections observed in areas distal to the lesion could be evidence of connectomal diaschisis [82], already introduced in stroke as changes in the functional and structural connectome in regions distant from the original lesion. Our research results explicitly showed a large global FC impairment in areas distal to the tumor location. This finding is consistent with the connectomic diaschisis hypothesized by Pasquini et al. [83]. In addition, FC specifically highlighted a high abnormality in the Right Central Visual, Right Peripheral Visual, Left Central Visual, and Left Peripheral Visual areas. Such anomalies could be linked to the neural responses as functional plasticity of visual processing induced by a brain tumor and investigated by Yang et al. [84].

It is also worth noting that the study evidenced generally higher SC impairment than FC changes in pathological tissue. On the contrary, a higher FC impairment, compared with SC affection, was observed in areas distal from the lesion. This finding confirmed that although glioma is classified as a tumor of the WM, it also affects distant functional regions. No other study has offered these complementary insights into the connectome. This outcome reinforces the infiltrative behavior of this brain tumor type, damaging predefined WM structures mostly connected to the lesion, causing functional disconnections near the lesion, but also spreading into distant functional cortical areas. Next, it is evident that FC dominates the FC + SC measure. The reason could be linked to internal functional changes starting before measurable anatomical damages. Further, FC changes could be derived from the dynamic remodeling of functional circuits [85] and necessarily be linked to functional variations. Finally, the lack of FC and SC coincident abnormalities out of the lesion could reflect the faster compensation/disconnection mechanism induced by FC, before a quantifiable WM structural damage, especially at the early onset of the pathology. It should be mentioned that FC anomaly does not necessarily indicate a disruptive behavior, but could also be the consequence of a compensatory change. It can also be hypothesized that networks showing distant functional changes could later become sites of tumor progression if the lesion damages the WM tracts connecting to those brain regions.

### 4.3 Limitations

The study also presents some limitations. The transfer learning procedure was applied to a reduced pool of oncological matrices; thus, even though selecting those with minimal impairments, it still presented glioma‐induced abnormalities. Next, low latent space dimensionality produces less precise reconstructions, but the value allows for later feature space analysis. Further, given the application of the SIFT [86] filtering approach for WM reconstruction tractography despite more recent state‐of‐the‐art recommendations, we could not study cortical–cortical inter‐hemispheric tracts and subcortical–subcortical intrahemispheric links. For rs‐fMRI and dMRI processing, the HCP data underwent slightly different steps compared with oncological imaging volumes. Nevertheless, the variational feature of the deep learning framework should overcome this issue. An additional limitation concerns the presence of high‐grade and low‐grade glioma in our dataset, also including not classified brain tumors. The glioma classification is based on WHO 2016 [37], due to the starting time of the patients' enrollment (2017). Hence, future steps could comprise the identification of a healthy dataset with protocol, scanner, and processing steps like the pathological one. Then, a larger and more homogeneous glioma dataset and the implementation of SIFT2 [87] WM tract filtering could improve the procedure. Further, novel diffusion approaches to diffusion connectomics, combining microstructure and tractography [88], may then be vital in the future to significantly control false positive pathways. In addition, anomaly detection procedure assessment on longitudinal acquisitions will be crucial to investigate the later spreading of the connectivity abnormalities, to check for later infiltrations and neuroplasticity phenomena. This could have an impact on glioma knowledge for clinical purposes. Finally, it is important to acknowledge that other anomaly detection methodologies could provide additional abnormalities beyond the proposed framework. Nonetheless, we intended to deliberately explore this integrated approach, ensuring the inclusion of all three sources of information throughout our study.

## 5 Conclusions

In summary, this study implemented a multimodal VAE architecture to detect anomalies in whole‐brain functional–structural connectome. The VAE framework effectively integrated complementary information from both FC and SC, enabling reliable reconstructions. The identified anomalies were observed in brain regions both near and far from the lesion site, which supports the idea that glioma affects distributed brain networks rather than just local structures.

## Author Contributions

M.Colpo and A.D.L. designed the study and performed the analysis. M.Colpo, A.D.L., and R.P. contributed to the interpretation of the data. M.Colpo and A.D.L. drafted the article. D.C. collected the data. M.Colpo, R.P., A.L., D.C., M.Corbetta, A.B., and A.D.L. reviewed the article and approved its final version.

## Funding

This work was supported by the Ministry of University and Research (MUR), National Recovery and Resilience Plan (NRRP), project MNESYS (PE0000006), Starting Grant from the European Research Council (101163214), Galen and Hilary Weston foundation, Stichting Hanarth Fonds, and Fondazione Ing. Aldo Gini scholarship.

## Conflicts of Interest

The authors declare no conflicts of interest.

## Supporting information


**Table S1:** Demographic and clinical information of oncological patients belonging to the test set. From the left, for each patient there are, demographics information, lesion histology, lesion type classification (WHO 2016^1^ classification), lesion hemisphere position, lesion lobe position, lesion volume and tumor volume. (Hemi = involved hemisphere, T + O volume = extent of the tumor + edema segmentation, T volume = extent of the tumor segmentation, CC = corpus callosum, F = frontal lobe, L = left, N.A. = not available, O = occipital lobe, P = parietal lobe, R = right, T = temporal lobe, WT = wild type, \ = not measured).
**Table S2:** Demographic and clinical information of oncological patients in the training and validation set of the transfer learning procedure. From the left, for each patient there are demographics information, lesion histology, lesion type classification (WHO 2016^1^ classification), lesion hemisphere position, lesion lobe position, lesion volume and tumor volume. (Hemi = involved hemisphere, T + O volume = extent of the tumor + edema segmentation, T volume = extent of the tumor segmentation, CC = corpus callosum, F = frontal lobe, L = left, N.A. = not available, O = occipital lobe, P = parietal lobe, R = right, T = temporal lobe, WT = wild type, \ = not measured).
**Figure S1:** Neural Network Architecture selected for the study.
**Figure S2:** Panel a) compares mean FC and mean FC reconstructed matrices, computed among the HCP subjects (after the power law transformation). Panel b) compares mean SC and mean SC reconstructed matrices, computed among the HCP subjects (after the power law transformation). Reconstructed matrices are obtained according to the selected procedure. VisCent = Visual Central network; VisPeri = Visual Peripheral network; SomMotA = Somatomotor‐A network; SomMotB = Somatomotor‐B network; DorsAttnA = Dorsal Attention‐A network; DorsAttnB = Dorsal Attention‐B network; SalVentAttnA = Salience/Ventral Attention‐A network; SalVentAttB = Salience/Ventral Attention‐B network; LimbicB = Limbic‐B network; LimbicA = Limbic‐A network; ControlA = Control‐A network; ControlB = Control‐B network; ControlC = Control‐C network; DefaultA = Default‐ A network; DefaultB = Default‐B network; DefaultC = Default‐C network; TempPar = Temporal Parietal network; Subcort = Subcortical network.
**Figure S3:** Panel a) compares mean FC and mean FC reconstructed matrices, computed among the HCP subjects (after the power law transformation). Panel b) compares mean SC and mean SC reconstructed matrices, computed among the HCP subjects (after the power law transformation). Reconstructed matrices are obtained providing as input a single FC or single SC matrix. VisCent = Visual Central network; VisPeri = Visual Peripheral network; SomMotA = Somatomotor‐A network; SomMotB = Somatomotor‐B network; DorsAttnA = Dorsal Attention‐ A network; DorsAttnB = Dorsal Attention‐B network; SalVentAttnA = Salience/Ventral Attention‐A network; SalVentAttB = Salience/Ventral Attention‐B network; LimbicB = Limbic‐B network; LimbicA = Limbic‐A network; ControlA = Control‐A network; ControlB = Control‐B network; ControlC = Control‐C network; DefaultA = Default‐A network; DefaultB = Default‐B network; DefaultC = Default‐C network; TempPar = Temporal Parietal network; Subcort = Subcortical network.
**Figure S4:** Violin plots representing MSSSIM, SSIM and MSE distributions, derived from the selected method (FC and SC concatenated as VAE input) and from the individual approach (FC and SC are singularly provided as VAE input). In Panel A there are violin plots concerning FC similarity measure distributions. In Panel B there are violin plots displaying SC similarity measure distributions.
**Figure S5:** Network Alteration Degree (NAD) derived from FC + SC, FC and SC for networks overlapping with different tissue types. Values associated with different connectivity modalities are displayed as illustrated in the legend. Patients are grouped according to lesion hemisphere position. From the top: patients with a lesion on the left hemisphere, patients with a lesion on the right hemisphere, patients with bilateral lesion. Results are presented for a threshold equal to thrHCP. Panel a): Network Alteration Degree derived from FC + SC, FC and SC for networks overlapping with the tumor core (T). Panel b): Network Alteration Degree derived from FC + SC, FC and SC for networks overlapping with the edema (O). Panel c): Network Alteration Degree derived from FC + SC, FC and SC for networks overlapping out of the pathological tissue. VisCent = Visual Central network; VisPeri = Visual Peripheral network; SomMotA = Somatomotor‐A network; SomMotB = Somatomotor‐B network; DorsAttnA = Dorsal Attention‐A network; DorsAttnB = Dorsal Attention‐B network; SalVentAttnA = Salience/Ventral Attention‐A network; SalVentAttB = Salience/Ventral Attention‐B network; LimbicB = Limbic‐B network; LimbicA = Limbic‐A network; ControlA = Control‐A network; ControlB = Control‐B network; ControlC = Control‐C network; DefaultA = Default‐A network; DefaultB = Default‐B network; DefaultC = Default‐C network; TempPar = Temporal Parietal network; Subcort = Subcortical network.
**Figure S6:** Overlap between Right Control‐B Network region and lesion(T + O)/tumor(T) frequency map (Panel a/b). Regions with the highest overlap in lesion(T + O)/tumor(T) occurrences are visualized in light yellow. The Right Control‐B Network is the region, overlapping with the edema (O), most frequently altered among the patients. Regions of the Right Control‐B Network are overlaid in bright green to visualize the overlap of patients' lesion(T + O)/tumor(T) distribution and network representation. Maps are superimposed on the MNI atlas (gray scale). Radiological convention.
**Figure S7:** Transfer learning impact was evaluated in terms of GD variations. For each subject and each modality, GD variations are evaluated and displayed. Panel a) refers to FC‐based variations, Panel b) refers to SC‐based variations, Panel c) refers to FC + SC‐based variations.
**Figure S8:** Transfer learning impact was evaluated in terms of ND variations. For each network and each modality, ND variations are evaluated and displayed. Panel a) refers to FC‐based variations, Panel b) refers to SC‐based variations, and Panel c) refers to FC + SC‐based variations. VisCent = Visual Central network; VisPeri = Visual Peripheral network; SomMotA = Somatomotor‐A network; SomMotB = Somatomotor‐B network; DorsAttnA = Dorsal Attention‐A network; DorsAttnB = Dorsal Attention‐B network; SalVentAttnA = Salience/Ventral Attention‐ A network; SalVentAttB = Salience/Ventral Attention‐B network; LimbicB = Limbic‐B network; LimbicA = Limbic‐A network; ControlA = Control‐A network; ControlB = Control‐B network; ControlC = Control‐C network; DefaultA = Default‐A network; DefaultB = Default‐B network; DefaultC = Default‐C network; TempPar = Temporal Parietal network; Subcort = Subcortical network.
**Figure S9:** tSNE visual representation of latent space features and k‐means clustering results. Panel a): tSNE representation of latent space features derived from HCP healthy data. K‐means provided 5 clusters that are displayed in the legend. Panel b): tSNE representation of latent space features derived from oncological test patients. K‐means provided 4 clusters that are displayed in the legend.
**Figure S10:** Varimax rotational technique outcomes applied on the first three principal components of a principal components analysis. On the right side there are results obtained from FC matrices, on the left side there are results obtained from SC matrices. Each column refers to each group individualized by the tSNE and k‐means procedure. Panel a): varimax results for HCP healthy data. Panel b): varimax results for oncological data. VisCent = Visual Central network; VisPeri = Visual Peripheral network; SomMotA = Somatomotor‐A network; SomMotB = Somatomotor‐B network; DorsAttnA = Dorsal Attention‐A network; DorsAttnB = Dorsal Attention‐B network; SalVentAttnA = Salience/Ventral Attention‐A network; SalVentAttB = Salience/Ventral Attention‐B network; LimbicB = Limbic‐B network; LimbicA = Limbic‐A network; ControlA = Control‐A network; ControlB = Control‐B network; ControlC = Control‐C network; DefaultA = Default‐A network; DefaultB = Default‐B network; DefaultC = Default‐C network; TempPar = Temporal Parietal network; Subcort = Subcortical network.
**Figure S11:** Surface brain representation highlighting parcels enhanced by the varimax procedure on HCP subjects. In Panel a) there are results obtained from FC matrices, in Panel b) there are results obtained from SC matrices. Each box outlines a different group.
**Figure S12:** Surface brain representation highlighting parcels enhanced by the varimax procedure on oncological subjects. In Panel a) there are results obtained from FC matrices, in Panel b) there are results obtained from SC matrices. Each box outlines a different group.

## Data Availability

The oncological data that support this study's findings are available from the corresponding author, upon reasonable request. The HCP‐A 2.0 Release data used in this report came from https://doi.org/10.15154/1520707. The codes and processed data that support the conclusions of this research work can be accessed via request to the corresponding author.

## References

[1] D. S. Bassett and O. Sporns , “Network Neuroscience,” Nature Neuroscience 20, no. 3 (2017): 353–364, 10.1038/nn.4502. 28230844 PMC5485642

[2] D. A. Maas and L. Douw , “Multiscale Network Neuroscience in Neuro‐Oncology: How Tumors, Brain Networks, and Behavior Connect Across Scales,” Neuro‐Oncology Practice 10, no. 6 (2023): 506–517, 10.1093/nop/npad044. 38026586 PMC10666814

[3] D. N. Louis , A. Perry , P. Wesseling , et al., “The 2021 WHO Classification of Tumors of the Central Nervous System: A Summary,” Neuro‐Oncology 23, no. 8 (2021): 1231–1251, 10.1093/neuonc/noab106. 34185076 PMC8328013

[4] M. J. van den Bent , M. Geurts , P. J. French , et al., “Primary Brain Tumours in Adults,” Lancet 402, no. 10412 (2023): 1564–1579, 10.1016/S0140-6736(23)01054-1. 37738997

[5] S. Lapointe , A. Perry , and N. A. Butowski , “Primary Brain Tumours in Adults,” Lancet 392, no. 10145 (2018): 432–446, 10.1016/S0140-6736(18)30990-5. 30060998

[6] Q. T. Ostrom , H. Gittleman , J. Fulop , et al., “CBTRUS Statistical Report: Primary Brain and Central Nervous System Tumors Diagnosed in the United States in 2008‐2012,” Neuro‐Oncology 17 (2015): iv1–iv62, 10.1093/neuonc/nov189. 26511214 PMC4623240

[7] L. R. Schaff and I. K. Mellinghoff , “Glioblastoma and Other Primary Brain Malignancies in Adults: A Review,” JAMA 329, no. 7 (2023): 574–587, 10.1001/jama.2023.0023. 36809318 PMC11445779

[8] V. A. Cuddapah , S. Robel , S. Watkins , and H. Sontheimer , “A Neurocentric Perspective on Glioma Invasion,” Nature Reviews. Neuroscience 15, no. 7 (2014): 455–465, 10.1038/nrn3765. 24946761 PMC5304245

[9] Y. Wei , C. Li , Z. Cui , et al., “Structural Connectome Quantifies Tumour Invasion and Predicts Survival in Glioblastoma Patients,” Brain 146, no. 4 (2023): 1714–1727, 10.1093/brain/awac360. 36189936 PMC10115235

[10] A. G. S. Daniel , K. Y. Park , J. L. Roland , et al., “Functional Connectivity Within Glioblastoma Impacts Overall Survival,” Neuro‐Oncology 23, no. 3 (2021): 412–421, 10.1093/neuonc/noaa189. 32789494 PMC7992880

[11] A. G. S. Daniel , C. D. Hacker , J. J. Lee , et al., “Homotopic Functional Connectivity Disruptions in Glioma Patients Are Associated With Tumor Malignancy and Overall Survival,” Neuro‐Oncology Advances 3, no. 1 (2021): vdab176, 10.1093/noajnl/vdab176. 34988455 PMC8694208

[12] S. D'Souza , L. Hirt , D. R. Ormond , and J. A. Thompson , “Retrospective Analysis of Hemispheric Structural Network Change as a Function of Location and Size of Glioma,” Brain Communications 3, no. 1 (2021): fcaa216, 10.1093/braincomms/fcaa216. 33501423 PMC7811759

[13] C. Magnon and H. Hondermarck , “The Neural Addiction of Cancer,” Nature Reviews. Cancer 23, no. 5 (2023): 317–334, 10.1038/s41568-023-00556-8. 37041409

[14] V. Venkataramani , D. I. Tanev , T. Kuner , W. Wick , and F. Winkler , “Synaptic Input to Brain Tumors: Clinical Implications,” Neuro‐Oncology 23, no. 1 (2021): 23–33, 10.1093/neuonc/noaa158. 32623467 PMC7850064

[15] R. Mancusi and M. Monje , “The Neuroscience of Cancer,” Nature 618, no. 7965 (2023): 467–479, 10.1038/s41586-023-05968-y. 37316719 PMC11146751

[16] F. Winkler , H. S. Venkatesh , M. Amit , et al., “Cancer Neuroscience: State of the Field, Emerging Directions,” Cell 186, no. 8 (2023): 1689–1707, 10.1016/j.cell.2023.02.002. 37059069 PMC10107403

[17] M. Monje , J. C. Borniger , N. J. D'Silva , et al., “Roadmap for the Emerging Field of Cancer Neuroscience,” Cell 181, no. 2 (2020): 219–222, 10.1016/j.cell.2020.03.034. 32302564 PMC7286095

[18] P. Hagmann , L. Cammoun , X. Gigandet , et al., “MR Connectomics: Principles and Challenges,” Journal of Neuroscience Methods 194, no. 1 (2010): 34–45, 10.1016/J.JNEUMETH.2010.01.014. 20096730

[19] F. Calamante , “The Seven Deadly Sins of Measuring Brain Structural Connectivity Using Diffusion MRI Streamlines Fibre‐Tracking,” Diagnostics 9 (2019): 15, 10.3390/diagnostics9030115. 31500098 PMC6787694

[20] R. E. Smith , J. D. Tournier , F. Calamante , and A. Connelly , “The Effects of SIFT on the Reproducibility and Biological Accuracy of the Structural Connectome,” NeuroImage 104 (2015): 253–265, 10.1016/J.NEUROIMAGE.2014.10.004. 25312774

[21] K. J. Friston , “Functional and Effective Connectivity: A Review,” Brain Connectivity 1, no. 1 (2011): 13–36, 10.1089/brain.2011.0008. 22432952

[22] P. H. Luckett , M. Olufawo , B. Lamichhane , et al., “Predicting Survival in Glioblastoma With Multimodal Neuroimaging and Machine Learning,” Journal of Neuro‐Oncology 164, no. 2 (2023): 309–320, 10.1007/s11060-023-04439-8. 37668941 PMC10522528

[23] K. Y. Park , A. Z. Snyder , M. Olufawo , et al., “Glioblastoma Induces Whole‐Brain Spectral Change in Resting State fMRI: Associations With Clinical Comorbidities and Overall Survival,” NeuroImage: Clinical 39 (2023): 103476, 10.1016/j.nicl.2023.103476. 37453204 PMC10371854

[24] B. Lamichhane , A. G. S. Daniel , J. J. Lee , D. S. Marcus , J. S. Shimony , and E. C. Leuthardt , “Machine Learning Analytics of Resting‐State Functional Connectivity Predicts Survival Outcomes of Glioblastoma Multiforme Patients,” Frontiers in Neurology 12 (2021): 642241, 10.3389/fneur.2021.642241. 33692747 PMC7937731

[25] G. Sprugnoli , L. Rigolo , M. Faria , et al., “Tumor BOLD Connectivity Profile Correlates With Glioma Patients' Survival,” Neuro‐Oncology Advances 4, no. 1 (2022): vdac153, 10.1093/noajnl/vdac153. 36532508 PMC9753902

[26] V. M. Stoecklein , S. Stoecklein , F. Galiè , et al., “Resting‐State fMRI Detects Alterations in Whole Brain Connectivity Related to Tumor Biology in Glioma Patients,” Neuro‐Oncology 22, no. 9 (2020): 1388–1398, 10.1093/neuonc/noaa044. 32107555 PMC7523460

[27] N. S. Dsouza , M. B. Nebel , D. Crocetti , J. Robinson , S. Mostofsky , and A. Venkataraman , “M‐GCN: A Multimodal Graph Convolutional Network to Integrate Functional and Structural Connectomics Data to Predict Multidimensional Phenotypic Characterizations,” in Proceedings of the Fourth Conference on Medical Imaging With Deep Learning, vol. 143, eds. M. Heinrich , Q. Dou , M. de Bruijne , J. Lellmann , A. Schläfer , and F. Ernst (PMLR, 2021), 119–130, https://proceedings.mlr.press/v143/dsouza21a.html.

[28] C. Doersch , “Tutorial on Variational Autoencoders,” Published Online June 19, 2016, http://arxiv.org/abs/1606.05908.

[29] A. Kebaili , J. Lapuyade‐Lahorgue , and S. Ruan , “Deep Learning Approaches for Data Augmentation in Medical Imaging: A Review,” Journal of Imaging 9, no. 4 (2023): 81, 10.3390/jimaging9040081. 37103232 PMC10144738

[30] C. Gong , C. Jing , X. Chen , et al., “Generative AI for Brain Image Computing and Brain Network Computing: A Review,” Frontiers in Neuroscience 17 (2023): 1203104, 10.3389/fnins.2023.1203104. 37383107 PMC10293625

[31] A. Hosny , C. Parmar , J. Quackenbush , L. H. Schwartz , and H. J. W. L. Aerts , “Artificial Intelligence in Radiology,” Nature Reviews Cancer 18, no. 8 (2018): 500–510, 10.1038/s41568-018-0016-5. 29777175 PMC6268174

[32] G. Martí‐Juan , M. Lorenzi , and G. Piella , “MC‐RVAE: Multi‐Channel Recurrent Variational Autoencoder for Multimodal Alzheimer's Disease Progression Modelling,” NeuroImage 268 (2023): 119892, 10.1016/j.neuroimage.2023.119892. 36682509

[33] E. Geenjaar , N. Lewis , Z. Fu , R. Venkatdas , S. Plis , and V. Calhoun , “Fusing Multimodal Neuroimaging Data With a Variational Autoencoder,” in Proceedings of the Annual International Conference of the IEEE Engineering in Medicine and Biology Society, EMBS, (Institute of Electrical and Electronics Engineers Inc., 2021), 3630–3633, 10.1109/EMBC46164.2021.9630806. 34892024

[34] E. P. T. Geenjaar , N. L. Lewis , A. Fedorov , et al., “Chromatic Fusion: Generative Multimodal Neuroimaging Data Fusion Provides Multi‐Informed Insights Into Schizophrenia,” Human Brain Mapping 44, no. 17 (2023): 5828–5845, 10.1002/hbm.26479. 37753705 PMC10619380

[35] A. Patcha and J. M. Park , “An Overview of Anomaly Detection Techniques: Existing Solutions and Latest Technological Trends,” Computer Networks 51, no. 12 (2007): 3448–3470, 10.1016/j.comnet.2007.02.001.

[36] M. P. Harms , L. H. Somerville , B. M. Ances , et al., “Extending the Human Connectome Project Across Ages: Imaging Protocols for the Lifespan Development and Aging Projects,” NeuroImage 183 (2018): 972–984, 10.1016/j.neuroimage.2018.09.060. 30261308 PMC6484842

[37] D. N. Louis , A. Perry , G. Reifenberger , et al., “The 2016 World Health Organization Classification of Tumors of the Central Nervous System: A Summary,” Acta Neuropathologica 131, no. 6 (2016): 803–820, 10.1007/s00401-016-1545-1. 27157931

[38] B. B. Avants , N. J. Tustison , G. Song , P. A. Cook , A. Klein , and J. C. Gee , “A Reproducible Evaluation of ANTs Similarity Metric Performance in Brain Image Registration,” NeuroImage 54, no. 3 (2011): 2033–2044, 10.1016/j.neuroimage.2010.09.025. 20851191 PMC3065962

[39] V. Fonov , A. C. Evans , K. Botteron , C. R. Almli , R. C. McKinstry , and D. L. Collins , “Unbiased Average Age‐Appropriate Atlases for Pediatric Studies,” NeuroImage 54, no. 1 (2011): 313–327, 10.1016/j.neuroimage.2010.07.033. 20656036 PMC2962759

[40] S. M. Smith , M. Jenkinson , M. W. Woolrich , et al., “Advances in Functional and Structural MR Image Analysis and Implementation as FSL,” NeuroImage 23 (2004): S208–S219, 10.1016/j.neuroimage.2004.07.051. 15501092

[41] M. F. Glasser , S. N. Sotiropoulos , J. A. Wilson , et al., “The Minimal Preprocessing Pipelines for the Human Connectome Project,” NeuroImage 80 (2013): 105–124, 10.1016/j.neuroimage.2013.04.127. 23668970 PMC3720813

[42] G. Salimi‐Khorshidi , G. Douaud , C. F. Beckmann , M. F. Glasser , L. Griffanti , and S. M. Smith , “Automatic Denoising of Functional MRI Data: Combining Independent Component Analysis and Hierarchical Fusion of Classifiers,” NeuroImage 90 (2014): 449–468, 10.1016/j.neuroimage.2013.11.046. 24389422 PMC4019210

[43] A. Schaefer , R. Kong , E. M. Gordon , et al., “Local‐Global Parcellation of the Human Cerebral Cortex From Intrinsic Functional Connectivity MRI,” Cerebral Cortex 28, no. 9 (2018): 3095–3114, 10.1093/cercor/bhx179. 28981612 PMC6095216

[44] B. T. Thomas Yeo , F. M. Krienen , J. Sepulcre , et al., “The Organization of the Human Cerebral Cortex Estimated by Intrinsic Functional Connectivity,” Journal of Neurophysiology 106, no. 3 (2011): 1125–1165, 10.1152/jn.00338.2011. 21653723 PMC3174820

[45] E. T. Rolls , C. C. Huang , C. P. Lin , J. Feng , and M. Joliot , “Automated Anatomical Labelling Atlas 3,” NeuroImage 206 (2020): 116189, 10.1016/j.neuroimage.2019.116189. 31521825

[46] A. S. Mandal , S. Brem , and J. Suckling , “Brain Network Mapping and Glioma Pathophysiology,” Brain Communications 5, no. 2 (2023): fcad040, 10.1093/braincomms/fcad040. 36895956 PMC9989143

[47] R. Romero‐Garcia , A. S. Mandal , R. A. I. Bethlehem , B. Crespo‐Facorro , M. G. Hart , and J. Suckling , “Transcriptomic and Connectomic Correlates of Differential Spatial Patterning Among Gliomas,” Brain 146, no. 3 (2023): 1200–1211, 10.1093/brain/awac378. 36256589 PMC9976966

[48] A. S. Mandal , R. Romero‐Garcia , M. G. Hart , and J. Suckling , “Genetic, Cellular, and Connectomic Characterization of the Brain Regions Commonly Plagued by Glioma,” Brain 143, no. 11 (2021): 3294–3307, 10.1093/BRAIN/AWAA277. PMC7891236 33278823

[49] A. S. Mandal , R. Romero‐Garcia , J. Seidlitz , M. G. Hart , A. F. Alexander‐Bloch , and J. Suckling , “Lesion Covariance Networks Reveal Proposed Origins and Pathways of Diffuse Gliomas,” Brain Communications 3, no. 4 (2021): fcab289, 10.1093/braincomms/fcab289. 34917940 PMC8669792

[50] C. De Luca , A. Virtuoso , M. Papa , F. Certo , G. M. V. Barbagallo , and R. Altieri , “Regional Development of Glioblastoma: The Anatomical Conundrum of Cancer Biology and Its Surgical Implication,” Cells 11, no. 8 (2022): 1349, 10.3390/cells11081349. 35456027 PMC9025763

[51] M. Jenkinson , C. F. Beckmann , T. E. J. Behrens , M. W. Woolrich , and S. M. Smith , “FSL,” NeuroImage 62, no. 2 (2012): 782–790, 10.1016/j.neuroimage.2011.09.015. 21979382

[52] O. Civier , M. Sourty , and F. Calamante , “MFCSC: Novel Method to Calculate Mismatch Between Functional and Structural Brain Connectomes, and Its Application for Detecting Hemispheric Functional Specialisations,” Scientific Reports 13, no. 1 (2023): 3485, 10.1038/s41598-022-17213-z. 36882426 PMC9992688

[53] D. P. Kingma and M. Welling , “Auto‐Encoding Variational Bayes,” Published Online December 20, 2013, http://arxiv.org/abs/1312.6114.

[54] I. Higgins , L. Matthey , A. Pal , et al., “β‐VAE: Learning Basic Visual Concepts With a Constrained Variational Framework,” (2022).

[55] D. P. Kingma and J. Ba , “Adam: A Method for Stochastic Optimization,” Published Online December 22, 2014, http://arxiv.org/abs/1412.6980.

[56] H. P. Chan , R. K. Samala , L. M. Hadjiiski , and C. Zhou , “Deep Learning in Medical Image Analysis,” in Deep Learning in Medical Image Analysis: Challenges and Applications, (Springer, 2020), 3–21, 10.1007/978-3-030-33128-3_1. PMC7442218 32030660

[57] M. Colpo , E. Silvestri , A. Salvalaggio , D. Cecchin , M. Corbetta , and A. Bertoldo , “Structural‐Functional Fingerprinting for Abnormalities Investigation in Glioma Patients,” Scientific Reports 15, no. 1 (2025): 38404, 10.1038/s41598-025-22192-y. 41184367 PMC12583605

[58] K. Weiss , T. M. Khoshgoftaar , and D. D. Wang , “A Survey of Transfer Learning,” Journal of Big Data 3, no. 1 (2016): 9, 10.1186/s40537-016-0043-6.

[59] B. C. M. van Wijk , C. J. Stam , and A. Daffertshofer , “Comparing Brain Networks of Different Size and Connectivity Density Using Graph Theory,” PLoS ONE 5, no. 10 (2010): e13701, 10.1371/journal.pone.0013701. 21060892 PMC2965659

[60] M. P. van den Heuvel , S. C. de Lange , A. Zalesky , C. Seguin , B. T. T. Yeo , and R. Schmidt , “Proportional Thresholding in Resting‐State fMRI Functional Connectivity Networks and Consequences for Patient‐Control Connectome Studies: Issues and Recommendations,” NeuroImage 152 (2017): 437–449, 10.1016/j.neuroimage.2017.02.005. 28167349

[61] B. M. de Brito Robalo , A. de Luca , C. Chen , et al., “Improved Sensitivity and Precision in Multicentre Diffusion MRI Network Analysis Using Thresholding and Harmonization,” NeuroImage: Clinical 36 (2022): 103217, 10.1016/j.nicl.2022.103217. 36240537 PMC9668636

[62] C. J. Honey , J. P. Thivierge , and O. Sporns , “Can Structure Predict Function in the Human Brain?,” NeuroImage 52, no. 3 (2010): 766–776, 10.1016/j.neuroimage.2010.01.071. 20116438

[63] C. J. Honey , O. Sporns , L. Cammoun , et al., “Predicting Human Resting‐State Functional Connectivity From Structural Connectivity,” Proceedings of the National Academy of Sciences of the United States of America 106, no. 6 (2009): 2035–2040, 10.1073/pnas.0811168106. 19188601 PMC2634800

[64] L. Wu and V. Calhoun , “Joint Connectivity Matrix Independent Component Analysis: Auto‐Linking of Structural and Functional Connectivities,” Human Brain Mapping 44, no. 4 (2023): 1533–1547, 10.1002/hbm.26155. 36420833 PMC9921228

[65] A. Abrol , Z. Fu , M. Salman , et al., “Deep Learning Encodes Robust Discriminative Neuroimaging Representations to Outperform Standard Machine Learning,” Nature Communications 12, no. 1 (2021): 353, 10.1038/s41467-020-20655-6. PMC7806588 33441557

[66] V. D. Calhoun and J. Sui , “Multimodal Fusion of Brain Imaging Data: A Key to Finding the Missing Link(S) in Complex Mental Illness,” Biological Psychiatry: Cognitive Neuroscience and Neuroimaging 1, no. 3 (2016): 230–244, 10.1016/j.bpsc.2015.12.005. 27347565 PMC4917230

[67] G. Rosenthal , F. Váša , A. Griffa , et al., “Mapping Higher‐Order Relations Between Brain Structure and Function With Embedded Vector Representations of Connectomes,” Nature Communications 9, no. 1 (2018): 2178, 10.1038/s41467-018-04614-w. PMC5988787 29872218

[68] A. Salvalaggio , L. Pini , A. Bertoldo , and M. Corbetta , “Glioblastoma and Brain Connectivity: The Need for a Paradigm Shift,” Lancet Neurology 23 (2024 http://www.thelancet.com/neurology): 740–748. 38876751 10.1016/S1474-4422(24)00160-1

[69] M. F. Hugues Duffau , “Moving Towards a Connectomic View of Neuro‐Oncology,” Lancet Neurology 23, no. 7 (2024): 655–656, 10.1016/S1474-4422(23)00291-0. 38876732

[70] V. Menon and M. D'Esposito , “The Role of PFC Networks in Cognitive Control and Executive Function,” Neuropsychopharmacology 47, no. 1 (2022): 90–103, 10.1038/s41386-021-01152-w. 34408276 PMC8616903

[71] S. Migliore , A. Ghazaryan , I. Simonelli , et al., “Validity of the Minimal Assessment of Cognitive Function in Multiple Sclerosis (MACFIMS) in the Italian Population,” Neurological Sciences 37, no. 8 (2016): 1261–1270, 10.1007/s10072-016-2578-x. 27095052

[72] A. Wallin , G. C. Román , M. Esiri , et al., “Update on Vascular Cognitive Impairment Associated With Subcortical Small‐Vessel Disease,” Journal of Alzheimer's Disease 62, no. 3 (2018): 1417–1441, 10.3233/JAD-170803. PMC5870030 29562536

[73] M. Ribeiro , Y. N. Yordanova , V. Noblet , G. Herbet , and D. Ricard , “White Matter Tracts and Executive Functions: A Review of Causal and Correlation Evidence,” Brain 147, no. 2 (2024): 352–371, 10.1093/brain/awad308. 37703295

[74] X. Zhang , G. Zhang , Y. Wang , et al., “Alteration of Default Mode Network: Association With Executive Dysfunction in Frontal Glioma Patients,” Journal of Neurosurgery 138, no. 6 (2023): 1512–1521, 10.3171/2022.8.JNS22591. 36242576

[75] J. Wang , M. Zhang , K. Yang and J. Chen , Synergistic Structural and Functional Alterations in the Medial Prefrontal Cortex of Patients With High‐Grade Gliomas Infiltrating the Thalamus and the Basal Ganglia (2023). 10.3389/fnins.2023.1136534 PMC10083262 37051149

[76] H. Zhang , Y. Shi , C. Yao , et al., “Alteration of the Intra‐ and Cross‐Hemisphere Posterior Default Mode Network in Frontal Lobe Glioma Patients,” Scientific Reports 6 (2016): 26972, 10.1038/srep26972. 27248706 PMC4888650

[77] K. Jütten , V. Mainz , D. Delev , et al., “Asymmetric Tumor‐Related Alterations of Network‐Specific Intrinsic Functional Connectivity in Glioma Patients,” Human Brain Mapping 41, no. 16 (2020): 4549–4561, 10.1002/hbm.25140. 32716597 PMC7555062

[78] K. Jütten , L. Weninger , V. Mainz , et al., “Dissociation of Structural and Functional Connectomic Coherence in Glioma Patients,” Scientific Reports 11, no. 1 (2021): 16790, 10.1038/s41598-021-95932-5. 34408195 PMC8373888

[79] A. Meyer‐Baese , K. Jütten , U. Meyer‐Baese , et al., “Controllability and Robustness of Functional and Structural Connectomic Networks in Glioma Patients,” Cancers (Basel) 15, no. 10 (2023): 2714, 10.3390/cancers15102714. 37345051 PMC10216571

[80] L. E. Suárez , R. D. Markello , R. F. Betzel , and B. Misic , “Linking Structure and Function in Macroscale Brain Networks,” Trends in Cognitive Sciences 24, no. 4 (2020): 302–315, 10.1016/j.tics.2020.01.008. 32160567

[81] D. H. Hadjiabadi , L. Pung , J. Zhang , et al., “Brain Tumors Disrupt the Resting‐State Connectome,” NeuroImage: Clinical 18 (2018): 279–289, 10.1016/j.nicl.2018.01.026. 29876248 PMC5987800

[82] E. Carrera and G. Tononi , “Diaschisis: Past, Present, Future,” Brain 137, no. 9 (2014): 2408–2422, 10.1093/brain/awu101. 24871646

[83] L. Pasquini , M. Jenabi , O. Yildirim , P. Silveira , K. K. Peck , and A. I. Holodny , “Brain Functional Connectivity in Low‐ and High‐Grade Gliomas: Differences in Network Dynamics Associated With Tumor Grade and Location,” Cancers 14, no. 14 (2022): 3327, 10.3390/cancers14143327. 35884387 PMC9324249

[84] J. Yang , N. Kudulaiti , Z. Chen , et al., “Within and Beyond the Visual Cortex: Brain Tumors Induce Highly Sensitive Plasticity of Visual Processing in Whole‐Brain Neural Functional Networks,” Cerebral Cortex 32, no. 20 (2022): 4422–4435, 10.1093/cercor/bhab492. 35106532

[85] S. Krishna and S. L. Hervey‐Jumper , “Neural Regulation of Cancer: Cancer‐Induced Remodeling of the Central Nervous System,” Advanced Biology 6, no. 9 (2022): 2200047, 10.1002/adbi.202200047. PMC10182823 35802914

[86] R. E. Smith , J. D. Tournier , F. Calamante , and A. Connelly , “SIFT: Spherical‐Deconvolution Informed Filtering of Tractograms,” NeuroImage 67 (2013): 298–312, 10.1016/j.neuroimage.2012.11.049. 23238430

[87] R. E. Smith , J. D. Tournier , F. Calamante , and A. Connelly , “SIFT2: Enabling Dense Quantitative Assessment of Brain White Matter Connectivity Using Streamlines Tractography,” NeuroImage 119 (2015): 338–351, 10.1016/j.neuroimage.2015.06.092. 26163802

[88] A. Daducci , A. Dal Palù , A. Lemkaddem , and J. P. Thiran , “COMMIT: Convex Optimization Modeling for Microstructure Informed Tractography,” IEEE Transactions on Medical Imaging 34, no. 1 (2015): 246–257, 10.1109/TMI.2014.2352414. 25167548

