# Supplementary material for: Anomaly Detection for Structural and Functional Connectivity in Glioma Patients
Source: NMR Biomed. 2026 Mar 10;39(4):e70238. doi: 10.1002/nbm.70238 (PMC12973340; doi:10.1002/nbm.70238)
Supplement: Supplementary file 1 — Table S1: Demographic and clinical information of oncological patients belonging to the test set. From the left, for each patient there are, demographics information, lesion histology, lesion type classification (WHO 20161 classification), lesion hemisphere position, lesion lobe position, lesion volume and tumor volume. (Hemi = involved hemisphere, T + O volume = extent of the tumor + edema segmentation, T volume = extent of the tumor segmentation, CC = corpus callosum, F = frontal lobe, L = left, N.A. = not available, O = occipital lobe, P = parietal lobe, R = right, T = temporal lobe, WT = wild type, \ = not measured). Table S2: Demographic and clinical information of oncological patients in the training and validation set of the transfer learning procedure. From the left, for each patient there are demographics information, lesion histology, lesion type classification (WHO 20161 classification), lesion hemisphere position, lesion lobe position, lesion volume and tumor volume. (Hemi = involved hemisphere, T + O volume = extent of the tumor + edema segmentation, T volume = extent of the tumor segmentation, CC = corpus callosum, F = frontal lobe, L = left, N.A. = not available, O = occipital lobe, P = parietal lobe, R = right, T = temporal lobe, WT = wild type, \ = not measured). Figure S1: Neural Network Architecture selected for the study. Figure S2: Panel a) compares mean FC and mean FC reconstructed matrices, computed among the HCP subjects (after the power law transformation). Panel b) compares mean SC and mean SC reconstructed matrices, computed among the HCP subjects (after the power law transformation). Reconstructed matrices are obtained according to the selected procedure. VisCent = Visual Central network; VisPeri = Visual Peripheral network; SomMotA = Somatomotor‐A network; SomMotB = Somatomotor‐B network; DorsAttnA = Dorsal Attention‐A network; DorsAttnB = Dorsal Attention‐B network; SalVentAttnA = Salience/Ventral Attention‐A network; SalVentAttB = Sali [file NBM-39-e70238-s001.pdf]

Anomaly Detection for Structural and Functional Connectivity in Glioma Patients

Maria Colpo[1,2,3], Ryan Pollitt[3], Alexander Leemans[3], Diego Cecchin[1,5], Maurizio Corbetta[1,4], Alessandra Bertoldo[1,2], Alberto De Luca[3,6]

[1] Padova Neuroscience Center, University of Padova, Padova, Italy,
[2] Department of Information Engineering, University of Padova, Padova, Italy,
[3] Image Sciences Institute, University Medical Center Utrecht, Utrecht, The Netherlands,
[4] Department of Neuroscience, University of Padova, Padova, Italy,
[5] Department of Medicine, Unit of Nuclear Medicine, University of Padova, Padova, Italy,
[6] Neurology Department, UMC Utrecht Brain Center, University Medical Center Utrecht, Utrecht, The Netherlands.

Supplementary Information:

1. Supplementary Material and Methods

Supplementary Table 1: Demographic and clinical information of oncological patients belonging to the test set. From the left, for each patient there are, demographics information, lesion histology, lesion type classification (WHO 2016[1] classification), lesion hemisphere position, lesion lobe position, lesion volume and tumor volume. (Hemi=involved hemisphere, T+O volume=extent of the tumor + oedema segmentation, T volume=extent of the tumor segmentation, CC= corpus callosum, F=frontal lobe, L=left, N.A.= not available, O=occipital lobe, P=parietal lobe, R=right, T=temporal lobe, WT=wild type, \=not measured).

Patient ID	Age	Gender	Histology	Classification	IDH1	Hemi	Lobe	T+O volume [cm3]	T volume [cm3]
1	25	F	Oligodendroglioma	Low grade	mutant	L	F	53,5	39,4
2	43	M	Glioblastoma	High grade	WT	L	F	81,1	0,4
3	69	M	Glioblastoma	High grade	WT	L	T	70,1	66,2
4	83	M	N.A.	N.A.	N.A.	L	P-T	11,7	10
5	67	F	Glioblastoma	High grade	WT	L	P	19,4	6,7
6	83	F	Glioblastoma epitelioid	High grade	WT	L	F-P	60,8	36,6
7	37	F	Astrocytoma	Low grade	mutant	L	F	11	11
8	56	M	Glioblastoma	High grade	mutant	L	F	127	88,8
9	75	F	Glioblastoma	High grade	WT	L	T	80,4	8,1
10	46	F	Glioneuronal neoplasm	High grade	mutant	L	F-insular	83,1	83,1
11	77	M	Glioblastoma	High grade	WT	L	T	103	85,4
12	57	M	Glioblastoma	High grade	WT	L	O-T	50,9	43,1
13	49	F	Glioblastoma	High grade	WT	L	T	36,8	34,5

14	54	F	Glioblastoma	High grade	WT	L	T	25,7	24,3
15	67	M	Glioblastoma	High grade	WT	L	F	49,3	48,1
16	50	M	Glioblastoma	High grade	N.A.	L	F	95,1	83,3
17	70	M	Glioblastoma	High grade	WT	L	T	18,9	16,3
18	58	F	Glioblastoma	High grade	WT	R	F-insular + splenium CC + P-O	76	73,9
19	42	M	Glioblastoma	High grade	mutant	R	F	139,2	123
20	61	M	Diffuse astrocytoma	Low grade	mutant	R	T	12,4	7,4
21	57	M	Glioblastoma	High grade	WT	R	F	191,6	108,3
22	83	M	N.A.	N.A.	N.A.	R	P-T-O	65,3	19
23	64	F	Glioblastoma	High grade	WT	R	T-P	155,9	155,9
24	73	M	Glioblastoma	High grade	WT	R	F	122,3	44,7
25	57	F	Glioblastoma	High grade	WT	R	F	112,4	99,5
26	64	F	Glioblastoma	High grade	WT	R	F	125,1	62,6
27	36	M	Glioblastoma	High grade	WT	B	F-CC	128,9	51,4
28	74	M	Diffuse large B-cell lymphoma	High grade	\	B	F-P + splenium CC	27,3	8,2
29	64	M	Glioblastoma	High grade	WT	B	F-insular R + CC L	67,9	54
30	68	M	Glioblastoma	High grade	WT	B	F-T-insular + cingulate cortex + splenium CC	134,2	127,5
31	73	M	N.A.	N.A.	N.A.	B	splenium CC	37,3	36,9

Supplementary Table 2: Demographic and clinical information of oncological patients in the training and validation set of the transfer learning procedure. From the left, for each patient there are demographics information, lesion histology, lesion type classification (WHO 2016[1] classification), lesion hemisphere position, lesion lobe position, lesion volume and tumor volume. (Hemi=involved hemisphere, T+O volume=extent of the tumor + oedema segmentation, T volume=extent of the tumor segmentation, CC= corpus callosum, F=frontal lobe, L=left, N.A.= not available, O=occipital lobe, P=parietal lobe, R=right, T=temporal lobe, WT=wild type, \=not measured).

**Patient ID**	**Age**	**Gender**	**Histology**	**Classification**	**IDH1**	**Hemi**	**Lobe**	**T+O volume [cm3]**	**T volume [cm3]**
T1	74	M	Diffuse glioneuronal tumor	High grade	\	L	T	30	5
T2	56	F	Intracranial mesenchymal tumor	Low grade	\	L	F	6,8	6,8
T3	74	F	Glioblastoma	High grade	WT	L	F	16,4	3,4
T4	56	F	Glioblastoma	High grade	WT	L	T	6,1	6,1
T5	49	M	Glioblastoma	High grade	WT	L	T	13,1	7,8
T6	32	F	Glioblastoma	High grade	WT	R	Thalamus	78,3	53,3

T7	48	F	Glioblastoma	High grade	WT	R	T + optic tract	56,4	53,4
T8	80	M	Glioblastoma	High grade	WT	R	O-T	17,7	17,3
T9	36	F	Not Otherwise Specified	Low grade	\	R	T	5,7	5,7
T10	51	M	Multinodular and vacuolating neuronal tumor	Low grade	\	R	P	14,9	14,9

1.1 Dataset1 - Protocol acquisition

The MRI acquisition protocol comprised a set of anatomical images, including two 3D T1-weighted (T1w) MPRAGE (TR/TE 2400/3.2 ms; voxel size 1x1x1 mm^3; FOV 256x256 mm^2; 160 slices), a 3D T2-weighted (T2w) FLAIR image (TR/TE 5000/395 ms; voxel size 1x1x1 mm^3; FOV 250x250 mm^2), acquired both before and after contrast agent injection and a T2w image (TR/TE 3200/536 ms; voxel size 1x1x1 mm^3; FOV 256x256 mm^2; 160 slices). In addition, a multi-shell dMRI protocol featured 100 volumes with 90 diffusion-weighted images (DWIs) (TR/TE 5355/104 ms; voxel size 2x2x2 mm^3; FOV 220x220 mm^2; 68 slices; multiband accelerator factor=2) divided in the following shells: 10 images at b=0 s/mm^2, 30 DWIs at b-value=710 s/mm^2 and 60 DWIs at b-value=2855 s/mm^2. The diffusion protocol applied was adjusted from the Zhang et al.[2] standard. Each diffusion direction was acquired with two reversed phase encoding directions, i.e., anterior-posterior and posterior-anterior directions, to later apply the distortion correction process next illustrated. In addition, rs-fMRI imaging included scans acquired with a T2*-weighted gradient-echo echo planar imaging (EPI) sequence (TR = 1260 ms, TE = 30 ms, FA = 68°, FOV = 204 × 204 mm2, voxel size = 3 × 3 × 3 mm3, iPAT = 0, multi-band acceleration factor (MBAccFactor) = 2, volumes = 750, TA = 16:03 min, phase encoding direction anteroposterior) and two spin echo-EPI acquisitions with reverse phase encoding (TR = 4200 ms, TE = 70 ms, FA = 90°, FOV = 204 × 204 mm2, voxel size = 3 × 3 × 3 mm3, TA = 8.4 s) for EPI distortion correction purposes.

1.2 Dataset2 - Protocol acquisition

HCP-Aging protocol acquisition[3] is summarized for convenience as follows. Structural T1-weighted and T2-weighted images were scanned with a multi-echo magnetization-prepared gradient-echo sequence. Bi-shell dMRI protocol featured four consecutive dMRI runs, sampled with 185 diffusion-weighting directions (92 DWIs at b-value=1500 s/mm^2 and 93 DWIs at b-value=3000 s/mm^2), each acquired twice with opposite phase encoding direction (AP and PA) to facilitate robust correction of distortions. 28 b-value=0 s/mm^2 volumes equally interspersed across the four runs (TR = 3230 ms; voxel size 1.5x1.5x1.5 mm3, MB = 4). Rs-fMRI images were collected using a 2D multiband gradient-recalled echo EPI sequence (TR = 800 ms; TE = 37 ms; flip angle = 52°; voxel size = 2.0 × 2.0 × 2.0 mm; volumes = 478; multiband factor = 8). Two sessions of eyes-open rs-fMRI (REST1 and REST2) were performed with opposite phase-encoding directions (four different runs of 6.5 min each). Additionally, a pair of opposite phase-encoding spin-echo were acquired to later correct functional images for signal distortion. Further details about the acquisition protocol and choices are reported in[3].

1.3 Dataset1 - Tumor segmentation and structural pre-processing

Each T1w image underwent structural preprocessing steps including bias field correction (*N4BiasFieldCorrection*[4]), skull-stripping (Multi-Atlas Skull Stripping[5]), and tissue segmentation (into GM, WM, and corticospinal fluid (CSF)) with the unified segmentation tool[6] of the Statistical Parametric Mapping, SPM12 v. 7771). Following these steps, the T1w image was separately normalized to both the symmetric MNI152 2009c atlas[7] and the symmetric MNI152 FSL atlas with a diffeomorphic non-linear registration (as implemented in ANTs SyN algorithm[78]). Following[9], MNI152 normalization was performed

excluding the tumoral lesion mask, to mitigate significant anatomical changes due to the tumor's presence in the alignment process. For each patient, the T and T + O masks were then mapped into the MNI152 FSL and MNI152 2009c exploiting the previously estimated diffeomorphic non-linear transformations. It should be specified that all the registration results underwent a visual quality check, to ensure anatomically accurate registrations.

1.4 Dataset1 - Diffusion imaging pre-processing and tractogram generation

dMRI volumes affected by interslice instabilities[10] (such as Venetian blinds artifacts[11]) were manually identified and removed, to guarantee better dMRI outcome processing. Several diffusion pre-processing steps were executed within the MRtrix3 software[12]. The pipeline sequence included: 1) random-matrix-based denoising via the *dwidenoise* command[13] and a B0-inhomogeneities joint correction, 2) subject motion, and 3) eddy currents via *dwifslpreproc*[14,15]. Finally, T1w segmentation images (including GM, subcortical parcellation, lesion, and tumor masks) were aligned to the naïve mean B0 volume using ANTs[16] software. An affine transformation, derived from the patient's original T1-weighted image, was a necessary step. This alignment was obtained through a single interpolation, derived from a transformation of the individual T1w image to the individual T2w image (via FSL's flirt registration[17]) and finally an affine transform (ANTs[16]) between the T2w and the B0 image. All resulting maps were visually inspected to ensure anatomical plausibility and accurate alignment with individual brain anatomy.

For each patient, tractography was generated as follows. At first, a multi-shell multi-tissue spherical deconvolution approach was performed to recover voxel-wise orientation distribution functions for each WM, GM, and CSF voxels[18]. Tractograms were subsequently reconstructed with 100M streamlines by employing Anatomically Constrained Tractography (ACT)[19]. Employing anatomical constraints, the individual brain tractogram was computed by utilizing a five-tissue-type (5TT) segmented tissue image, derived from the patient's structural T1-weighted image through the MRtrix *5ttgen* routine. Notably, the fifth tissue, representing pathological tissue, was incorporated into the segmentation by manually adding the lesion mask outlined by the expert neuroradiologist. WM-GM interface was the seeding area. Tractogram streamlines reconstruction was performed employing a second-order Integration over Fiber Orientation Distributions (iFOD2) algorithm[20]. Streamlines termination criteria were set as recommended default (maximum angle $\alpha = 45°$, FOD amplitude cut-off value of 0.1). Eventually, to reduce the impact of false positives, the 100M structural connectomes were quantitatively reduced to 10M using the Spherical-deconvolution Informed Filtering of Tractograms framework[21] (SIFT), by pruning off unreliable streamlines.

1.5 Dataset1 - Functional imaging pre-processing

rs-fMRI data underwent standard pre-processing featuring slice timing[22], readout distortion (FSL's TOPUP[15]) and motion (FSL's *mcflirt*) correction, a non-linear mapping to the symmetric MNI152 2009c atlas[7] through the subject-specific T1w (via FSL's boundary-based registration[23]) and high pass filtering (cut-off frequency: 0.008 Hz). It has to be noted that all the registration results were checked visually to ensure they met the anatomy. Group's independent component *analysis toolbox* (*GIFT*) toolbox (http://trendscenter.org/software/gift/) was later applied to decompose the functional pre-processed signals, all brought in the MNI152 2009c atlas[7] space. At first, scanner artifacts' volumes were regressed out from the patients' pre-processed images using an independent component approach (ICA)[24]. Then, the independent components (ICs) related to sequence MB factor or head movement artifacts were manually selected and regressed out from the rs-fMRI pre-processed data[25,26]. Additional information regarding the

selection process of ICs can be found in[27]. Then, 10 principal components related to CSF and WM signal (5 from WM, 5 from CSF) were regressed out from the rs-fMRI time series. This regression process also included the removal of the six standard head motion parameters[28]. The next step included a low pass filter (cut-off frequency: 0.11 Hz). Finally, to quantify the subject-specific head motion during the scan, frame-wise displacement was derived as defined in[29]. Eventually, subjects affected by high head motion (less than 400 volumes with mean FD<0.4 mm) were discarded.

1.6 Dataset2 - Functional imaging processing

Functional signals underwent a first high pass filtering (cut-off frequency: 0.008 Hz). Then, 10 principal components related to CSF and WM signal (5 from WM, 5 from CSF) were regressed out from the rs-fMRI time series. The removal of six standard head motion parameters was also included in the regression process[28]. The next operation included a low pass filter (cut-off frequency: 0.11 Hz). In addition, frame-wise displacement[29] was derived to quantify the subject-specific head motion during the scan. Eventually, subjects' runs affected by high rs-fMRI head motion (less than 263 volumes with mean FD<0.4 mm) were discarded. 155 subjects had the requisites just described.

1.7 Variational Autoencoder

Architecture:

Reconstruction quality assessment was evaluated through three similarity methods commonly used for structural similarity measurement[30,31]: mean squared error (MSE)[32], similarity structural index (SSIM)[33], and multiscale structural similarity index (MSSSIM)[34]. MSE varies in the range [0,1], where the lower the value, the better the reconstruction. SSIM and MSSSIM values are within the range [0,1], where the higher the value, the better the reconstruction.

For the sake of clarity, different VAE architectures were evaluated for the project. The encoder nodes number was increased to 4800, 2400, and 1200. Symmetrically, the decoder was composed of layers of 1200, 2400, and 4800 nodes. The latent space dimension was defined as equal to 600 nodes. This VAE framework was not later implemented in the project for the reasons explained in the discussion.

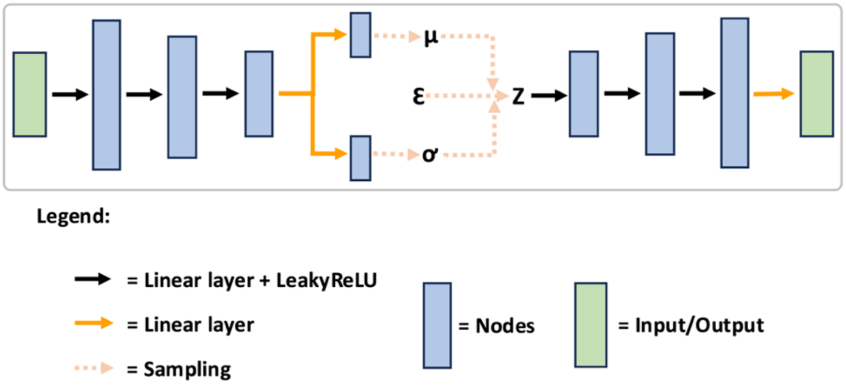

Supplementary Figure 1: Neural Network Architecture selected for the study.

1.8 Oncological Transfer Learning

Fine-tuning was performed on selected glioma patients with limited abnormalities. These glioma subjects were chosen based on preliminary results of different study, which investigated the SC and FC alterations and their interplay in brain tumors. Subjects selected for fine-tuning training and validation presented a

lower alteration impact and patient lesion positions were equally distributed between the left and right hemispheres. Thus, transfer learning was applied to 7 selected glioma patients. A later validation was performed on the other 3 chosen brain tumor patients. Criteria applied were identical to HCP neural network training, just lowering the lr to $1e^{-4}$, as suggested in[35]. Finally, the test was implemented on the remaining 31 subjects, obtaining a reconstructed FC_{recon} and SC_{recon} connectivity matrices for each individual. As outlined for healthy subjects, the model provided the inverse hyperbolic tangent version of FC_{recon} and SC_{recon} reconstructed. For this reason, final reconstructed matrices were derived after the employment of a hyperbolic tangent. To take into account that SC_{recon} was the transformed version of the SC matrix, resulting from the application of the Civier procedure[36]. Some SC_{recon} values were slightly negative and were therefore set to 0.

1.9 Anomaly detection

Considering the test HCP data, for each subject FC and SC connectivity matrices were reconstructed as described in the VAE architecture description. Thus, for both connectivity modalities, the absolute differences between the original FC and its reconstructed FC_{recon} and novel SC and its SC_{recon} were computed, obtaining the diffFC and diffSC matrices. Then, all diffFC and diffSC matrices were masked by the $meanFC\_80percent_{HCP}$ and $meanSC\_80percent_{HCP}$ matrices, to retain higher edge weights and to generate diffFC_mask and diffSC_mask matrices. Finally, z-score standardization was applied on upper triangular diffFC_mask and diffSC_mask matrices, reconstructing the square format and creating diffFC_mask_zscore and diffSC_mask_zscore matrices, to ensure the comparability of the two distributions. To infer an integrated connectivity measure, every single subject diffFC_mask_zscore and diffSC_mask_zscore matrices were summed, to derive the diffFC+SC_mask_zscore matrix. The process is illustrated in Panel b) of Figure 1. For each subject and each row (thus for each parcel) of diffFC_mask_zscore, diffSC_mask_zscore, and diffFC+SC_mask_zscore matrices, the average values were assessed. Accordingly, each single subject's parcel was represented by a single mean value indicated with the general label Par_{val}.

Then, for each connectivity modality, the distribution of parcels' mean values (Par_{val}) across all HCP test subjects was plotted. Due to the quite normal distribution, the threshold (thr_{HCP}) was defined as the mean value plus three standard deviations (mean+3SD). thr_{HCP} provided a balance between capturing outliers, values that were significantly different from most of the data, and maintaining a reasonable level of sensitivity to variability in the data.

Oncological test patients FC and SC matrices underwent reconstruction's steps following the same procedure described in the Oncological Transfer Learning section. It is worth noting that diffFC and diffSC glioma matrices were masked with $meanFC\_80percent_{HCP}$ and $meanSC\_80percent_{HCP}$ matrices. Visual representation of these steps is illustrated in Panel b) of Figure 1.

In right Panel c) of Figure 1, the strategy for defining altered connectivity measures for oncological patients is outlined. At this point, for each connectivity modality, the distribution of parcels' mean values across the patients was considered. Parcels' values (Par_{val}) were labeled as abnormal if they belonged to the right tail of the distribution. The threshold was chosen equal to thr_{HCP}. This selection effectively identified deviations that were indicative of pathology, being likely to have high sensitivity and specificity in detecting glioma changes, as they were calibrated against the healthy control range. Thus, for each patient, a parcel was defined as potentially altered if the following condition was true:

$$Par_{val}(i,j) > \mathrm{thr}_{\mathrm{HCP}}$$

Equation 1

where (i,j) defines the specific parcel i and patient j examined.

Eventually, for each connectivity modality, a vector (i.e., the vector of the impaired parcels) containing all the parcels that were found to be potentially altered was created. Vectors corresponding to the analyzed patient were arranged side by side to create a unified matrix with dimensions of 210x31.

1.10 Fine-Tuning impact

At first, the fine-tuning impact was evaluated in terms of Global Degree (GD) variations. Indicating with $GD_{NO\text{-}TL}$ the oncological global disruption derived from the anomaly detection procedure on HCP model parameters, and with GD the global degree derived from the selected procedure, for each subject and each modality, GD variations (GD_{var}) were evaluated as:

$$GD_{var} = \frac{GD_{NO-TL} - GD}{GD_{NO-TL}} \cdot 100$$

Equation 2

Moreover, the transfer learning impact on brain networks was investigated. Indicating with total network disruption (tND) the number of altered subjects within each network, and referring to $tND_{NO\text{-}TL}$ as the network disruption derived from the anomaly detection on the no transfer learning results. For each network and for each modality, tND variations (tND_{var}) were evaluated as:

$$tND_{var} = \frac{tND_{NO-TL} - tND}{tND_{NO-TL}} \cdot 100$$

Equation 3

1.11 Exploratory latent-space characterization (non-inferential)

In this exploratory analysis, the low-dimensional structure learned by the VAE was examined to describe which connectivity components (parcels/networks) contribute most to the latent representation of concatenated FC+SC. The aim was about identifying patients' grouping connections. Latent vectors were first visualized using t-distributed Stochastic Neighbor Embedding (t-SNE) to obtain a two-dimensional map for qualitative inspection[37]. Subjects were grouped in t-SNE space using k-means, and for each partition a mean connectivity matrix (210×210) was computed. Principal component analysis (PCA) with varimax rotation was then applied to the partition-specific mean matrices, and the absolute rotated loadings from the first three principal components were concatenated to form a parcels-by-partitions matrix (210×#partitions). We expect each cluster to represent the key connectivity features that explain the most variance in latent space, and not necessarily to correspond to any clinical subgroup. To facilitate interpretation, features with absolute rotated loadings above 0.12 (chosen empirically from the loading distribution to balance sparsity and readability) were highlighted as "most informative," indicating strong contribution to explain variance in the latent representation. The same procedure was applied to the original oncological matrices for descriptive comparison. This material is intended solely to characterize the composition of the latent space and its variance. In its current form, it is not used for inference and does not aim to define clinically meaningful subgroups.

2. Supplementary Results

2.1 Unraveling HCP connectivity reconstruction with VAE

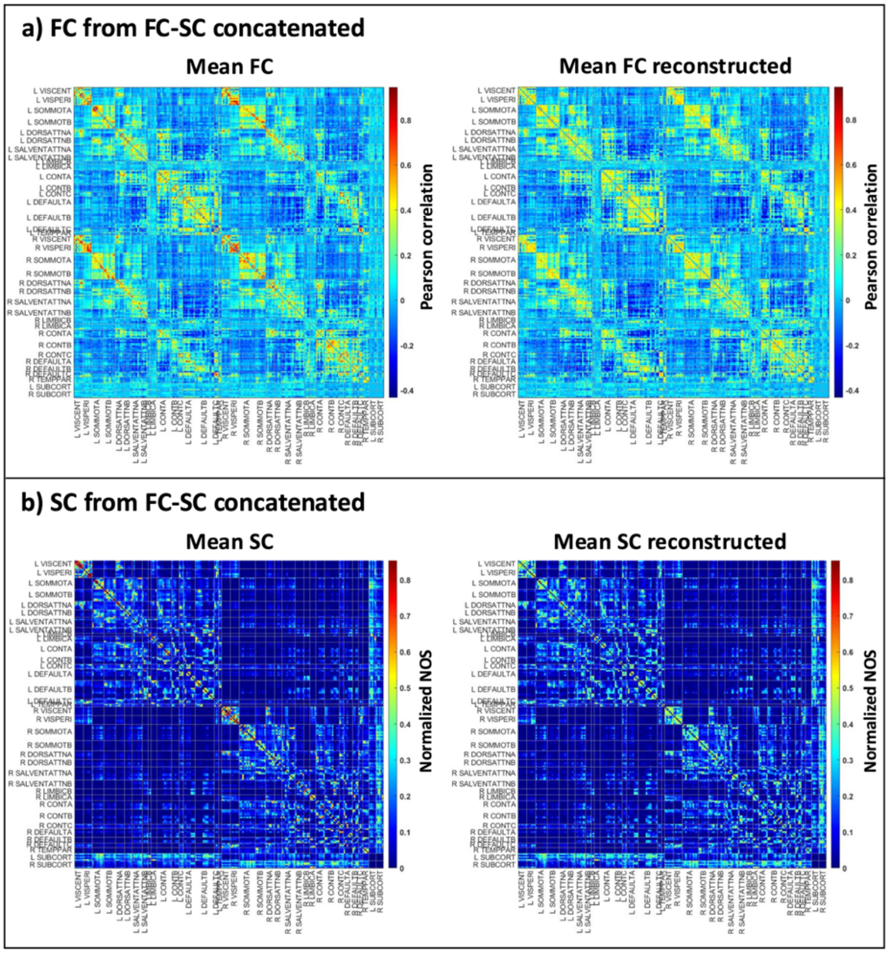

Supplementary Figure 2: Panel a) compares mean FC and mean FC reconstructed matrices, computed among the HCP subjects (after the power law transformation). Panel b) compares mean SC and mean SC reconstructed matrices, computed among the HCP subjects (after the power law transformation). Reconstructed matrices are obtained according to the selected procedure. VisCent = Visual Central network; VisPeri = Visual Peripheral network; SomMotA = Somatomotor-A network; SomMotB = Somatomotor-B network; DorsAttnA = Dorsal Attention-A network; DorsAttnB = Dorsal Attention-B network; SalVentAttnA = Salience/Ventral Attention-A network; SalVentAttB = Salience/Ventral Attention-B network; LimbicB = Limbic-B network; LimbicA = Limbic-A network; ControlA = Control-A network; ControlB = Control-B network; ControlC = Control-C network; DefaultA = Default-A network; DefaultB = Default-B network; DefaultC = Default-C network; TempPar = Temporal Parietal network; Subcort = Subcortical network.

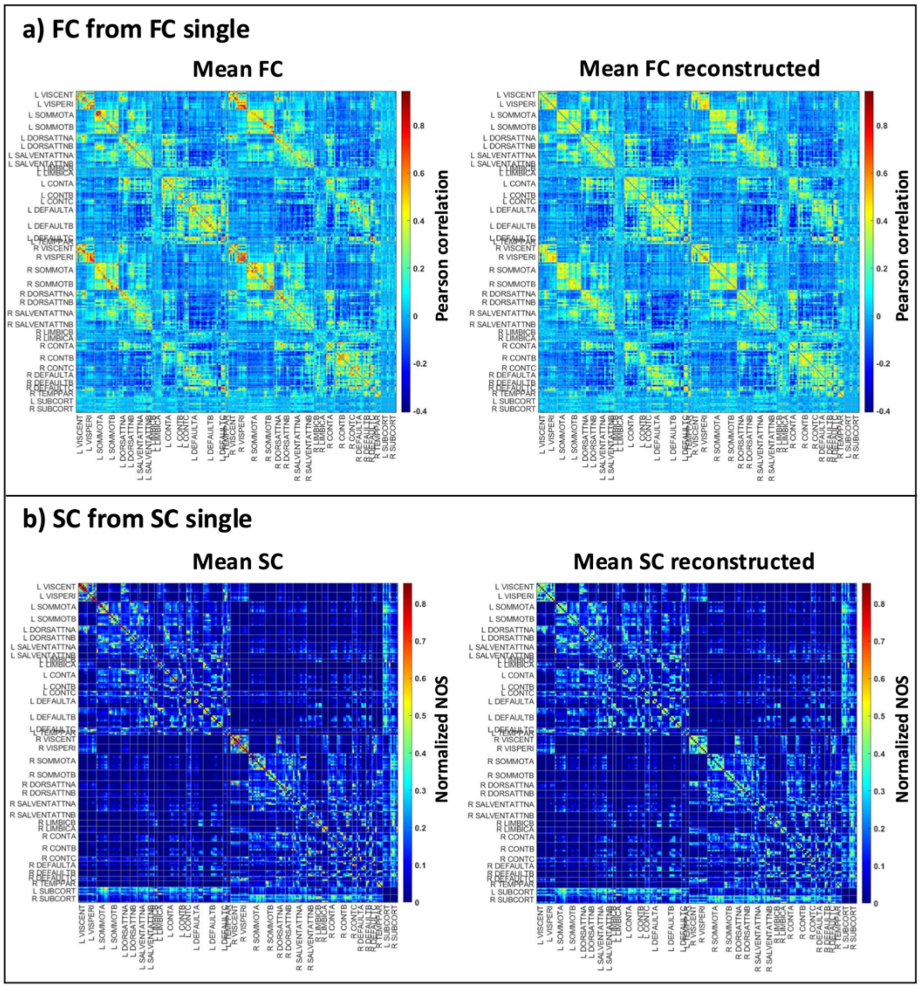

Supplementary Figure 3: Panel a) compares mean FC and mean FC reconstructed matrices, computed among the HCP subjects (after the power law transformation). Panel b) compares mean SC and mean SC reconstructed matrices, computed among the HCP subjects (after the power law transformation). Reconstructed matrices are obtained providing as input a single FC or single SC matrix. VisCent = Visual Central network; VisPeri = Visual Peripheral network; SomMotA = Somatomotor-A network; SomMotB = Somatomotor-B network; DorsAttnA = Dorsal Attention-A network; DorsAttnB = Dorsal Attention-B network; SalVentAttnA = Salience/Ventral Attention-A network; SalVentAttB = Salience/Ventral Attention-B network; LimbicB = Limbic-B network; LimbicA = Limbic-A network; ControlA = Control-A network; ControlB = Control-B network; ControlC = Control-C network; DefaultA = Default-A network; DefaultB = Default-B network; DefaultC = Default-C network; TempPar = Temporal Parietal network; Subcort = Subcortical network.

A second significant outcome was the examination of similarity measures. Reconstruction quality was assessed between single-subject FC and FC_{recon} and single-subject SC and SC_{recon}. Violin plots of Supplementary Figure 4 present the MSE, SSIM, and MSSSIM distributions, derived from the selected method (FC and SC concatenated as VAE input) and from the individual approach (FC and SC are singularly provided as VAE input). Considering that reconstruction accuracy cares about MSE minimization and SSIM and MSSIM maximization, Supplementary Figure 4 highlights a generally better performance of the concatenated predefined method compared to the single-input approach. Results were confirmed by two groups of Wilcoxon's matched pairs signed rank test. This crossover test compared MSE, SSIM, and MSSSIM distributions between six couples, resulting in a statistical significance (p-value<0.05) for all (both with Bonferroni and FDR correction).

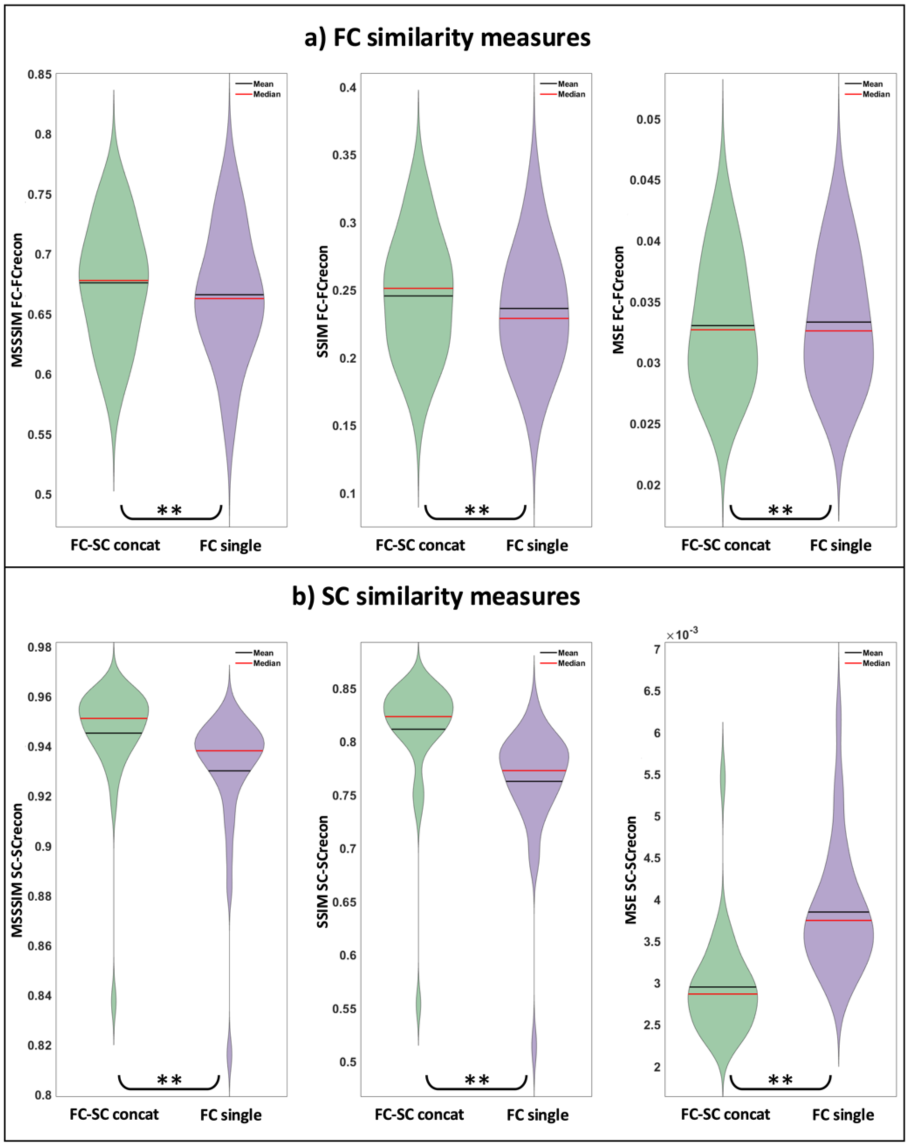

Supplementary Figure 4: Violin plots representing MSSSIM, SSIM and MSE distributions, derived from the selected method (FC and SC concatenated as VAE input) and from the individual approach (FC and SC are singularly provided as VAE input). In Panel A there are violin plots concerning FC similarity measure distributions. In Panel B there are violin plots displaying SC similarity measure distributions.

Regarding the VAE architecture tested in addition to the one selected for the work, slightly better MSE train loss and MSE validation loss were obtained (respectively equivalent to 0.0179 and 0.0187). Nonetheless, this tiny improvement did not justify such a higher node number, especially in the latent space dimension, which complicates the analysis of features in the latent space. From a reconstruction point of view, Spearman correlation values were again computed between meanFC and $meanFC_{recon}$ and meanSC and $meanSC_{recon}$, with values respectively equivalent to $Rho = 0.915$ (p-value<0.05) and $Rho = 0.855$ (p-value<0.05). Correlation values are lower than outcomes obtained from the selected VAE architecture.

2.2 Global connectivity impairments

In connection with GD_{FC}, tumoral (T) volume and lesion (T+O) volume exhibited positive correlations with GD_{FC} measure of networks in overlap with the tumor (Pearson correlation coefficients: $r = 0.49$, *p-value* < 0.05 and $r = 0.52$, *p-value* < 0.05, respectively (FDR corrected)). A positive correlation was also observed between GD_{FC} of networks in overlay with lesion and lesion (T+O) volume (Pearson correlation coefficients: $r = 0.5$, *p-value* < 0.05 (FDR corrected)). Moreover, tumor (T) volume and lesion (T+O) volume exhibited positive correlations with GD_{SC} measure (Pearson correlation coefficients: $r = 0.48$, *p-value* < 0.05 and $r = 0.75$, *p-value* < 0.05, respectively (FDR corrected)). The positive correlation became even stronger if considering the relationship between tumor (T) or lesion (T+O) volume and GD_{SC} linked to networks overlapping with tumor (T) and with lesion (T+O) (Pearson correlation coefficients: $r = 0.71$, *p-value* < 0.05 and $r = 0.62$, *p-value* < 0.05, $r = 0.45$, *p-value* < 0.05 and $r = 0.73$, *p-value* < 0.05, respectively (FDR corrected)). Eventually, the Pearson correlation between lesion (T+O) volume and GD_{SC} derived by networks overlapping with oedema (O) featured a good value (Pearson correlation: $r = 0.4$, *p-value* < 0.05 (FDR corrected)). Similar outcomes were obtained about $GD_{\underline{FC+SC}}$. Tumor (T) volume and lesion (T+O) volume exhibited positive correlations with $GD_{\underline{FC+SC}}$ measure (Pearson correlation coefficients: $r = 0.46$, *p-value* < 0.05 and $r = 0.71$, *p-value* < 0.05, respectively (FDR corrected)). The positive correlation became even stronger if considering the relationship between tumor (T) or lesion (T+O) volume and $GD_{\underline{FC+SC}}$ linked to networks overlapping with tumor (T) and with lesion (T+O) (Pearson correlation coefficients: $r = 0.66$, *p-value* < 0.05 and $r = 0.67$, *p-value* < 0.05, $r = 0.55$, *p-value* < 0.05 and $r = 0.75$, *p-value* < 0.05, respectively (FDR corrected)). The Pearson correlation between lesion (T+O) volume and $GD_{\underline{FC+SC}}$ derived by networks overlapping with oedema (O) also featured a positive value (Pearson correlation: $r = 0.44$, *p-value* < 0.05 (FDR corrected)).

OI_{SC} demonstrated good correlations with tumoral (T) and lesional (T+O) volumes (Spearman correlation coefficients: $Rho = 0.53$, *p-value* < 0.05 and $Rho = 0.75$, *p-value* < 0.05 respectively (FDR corrected)). Positive correlation results were also obtained between OI_{SC} of networks in overlay with tumor (T) and lesion (T+O) and tumoral (T) and lesional (T+O) volumes (Spearman correlation coefficients: $Rho = 0.56$, *p-value* < 0.05 and $Rho = 0.51$, *p-value* < 0.05, $Rho = 0.55$, *p-value* < 0.05 and $Rho = 0.47$, *p-value* < 0.05 respectively (FDR corrected)). Finally, a tiny relation was obtained between OI_{SC} of networks on the overlay with oedema (O) and lesional (T+O) volume (Spearman correlation coefficients: $Rho = 0.39$, *p-value* < 0.05).

Supplementary Figure 5 show the network alteration degree (NAD) derived for each patient. Generally, for each connectivity modality, patients with lesions in either the left or right hemisphere exhibited altered networks within the affected hemisphere, but also in the contralateral hemisphere. Bilateral patients demonstrated altered networks in both hemispheres.

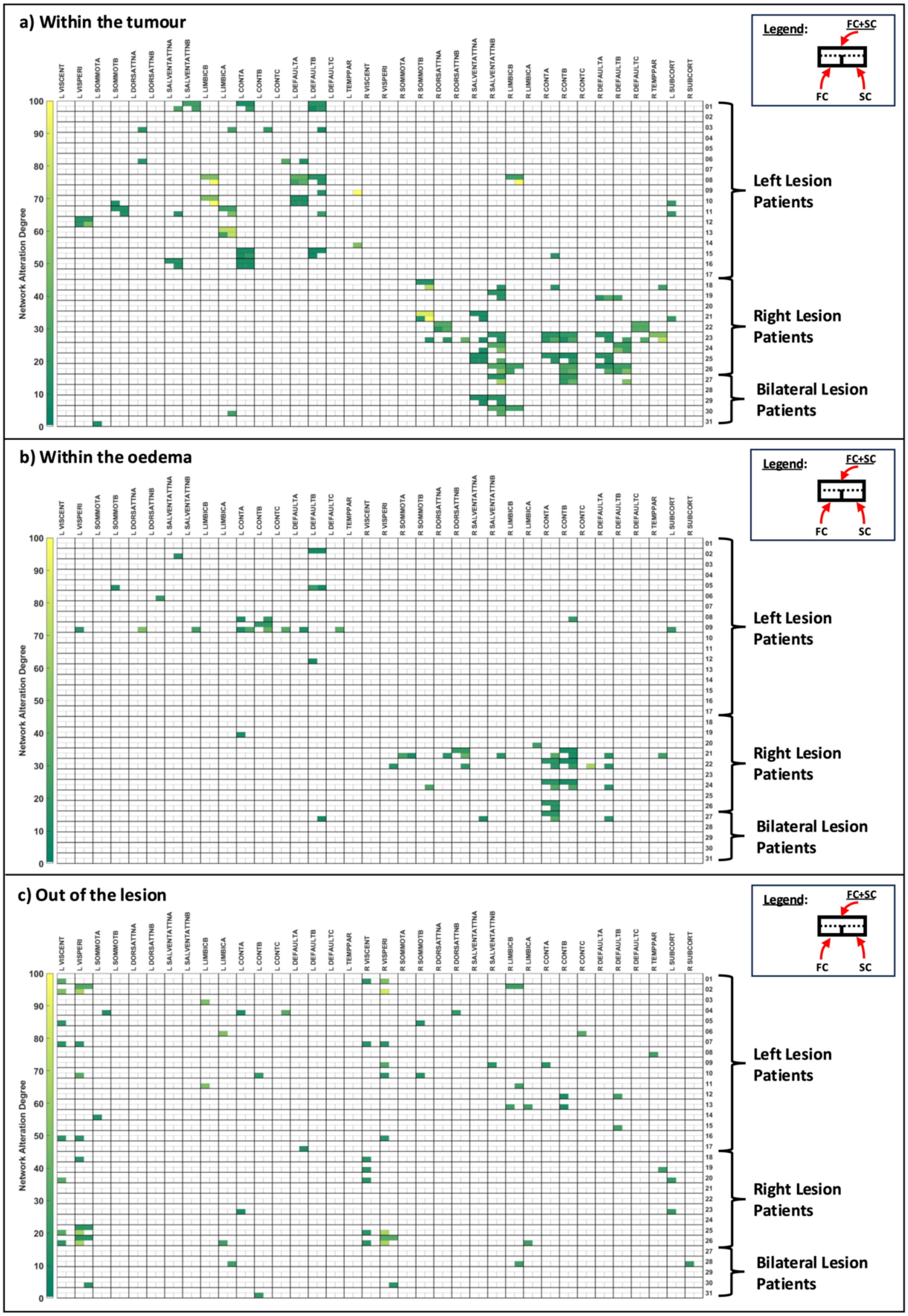

Supplementary Figure 5: Network Alteration Degree (NAD) derived from FC+SC, FC and SC for networks overlapping with different tissue types. Values associated with different connectivity modalities are displayed as illustrated in the legend. Patients are grouped according to lesion hemisphere position. From the top: patients with a lesion on the left hemisphere, patients with a lesion on the right hemisphere, patients with bilateral lesion. Results are presented for a threshold equal to thr$_{HCP}$. Panel a): Network Alteration Degree derived from FC+SC, FC and SC for

networks overlapping with the tumor core (T). Panel b): Network Alteration Degree derived from <u>FC+SC</u>, FC and SC for networks overlapping with the oedema (O). Panel c): Network Alteration Degree derived from <u>FC+SC</u>, FC and SC for networks overlapping out of the pathological tissue. VisCent = Visual Central network; VisPeri = Visual Peripheral network; SomMotA = Somatomotor-A network; SomMotB = Somatomotor-B network; DorsAttnA = Dorsal Attention-A network; DorsAttnB = Dorsal Attention-B network; SalVentAttnA = Salience/Ventral Attention-A network; SalVentAttB = Salience/Ventral Attention-B network; LimbicB = Limbic-B network; LimbicA = Limbic-A network; ControlA = Control-A network; ControlB = Control-B network; ControlC = Control-C network; DefaultA = Default-A network; DefaultB = Default-B network; DefaultC = Default-C network; TempPar = Temporal Parietal network; Subcort = Subcortical network.

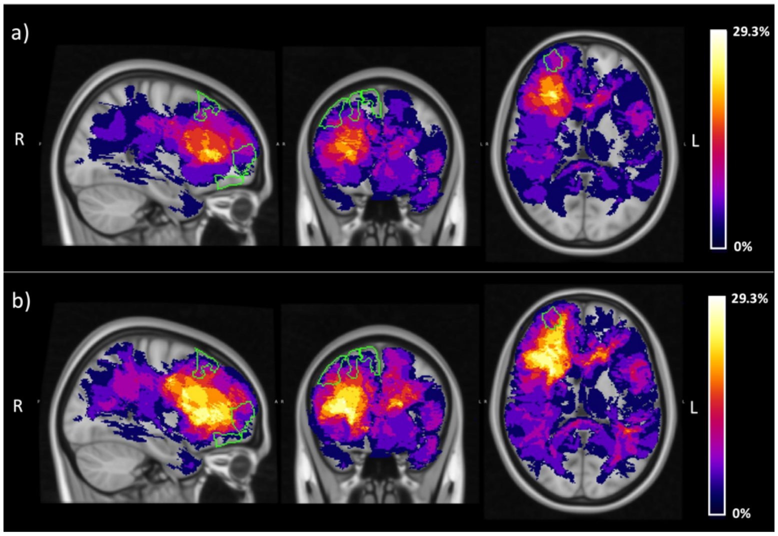

Supplementary Figure 6: Overlap between Right Control-B Network region and lesion(T+O)/tumor(T) frequency map (Panel a/b). Regions with the highest overlap in lesion(T+O)/tumor(T) occurrences are visualized in light yellow. The Right Control-B Network is the region, overlapping with the oedema (O), most frequently altered among the patients. Regions of the Right Control-B Network are overlaid in bright green to visualize the overlap of patients' lesion(T+O)/tumor(T) distribution and network representation. Maps are superimposed on the MNI atlas (grey scale). Radiological convention.

2.3 Investigating the relationships of single and integrated connectivity

Transfer learning impact was assessed both in terms of GD_{var} and ND_{var}. Supplementary Fig. 7 provides an overview of the variability among the subjects. Patients show different behaviors depending on the considered modality, thus with transfer learning (choice selected for the study) and without transfer learning. Investigating the network variations, Supplementary Fig. 8 displays the variations across the networks. Dorsal Attention, Salience/Ventral Attention, Control, and Default seem to be the more variable networks comparing the two approaches.

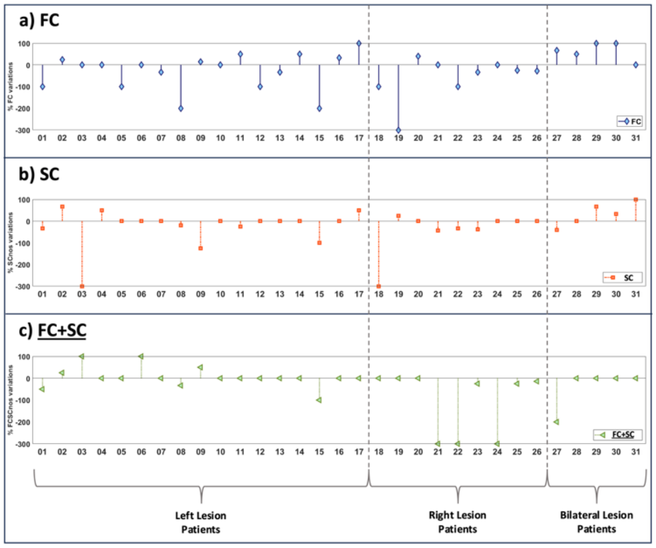

Supplementary Figure 7: Transfer learning impact was evaluated in terms of GD variations. For each subject and each modality, GD variations are evaluated and displayed. Panel a) refers to FC-based variations, Panel b) refers to SC-based variations, Panel c) refers to FC+SC-based variations.

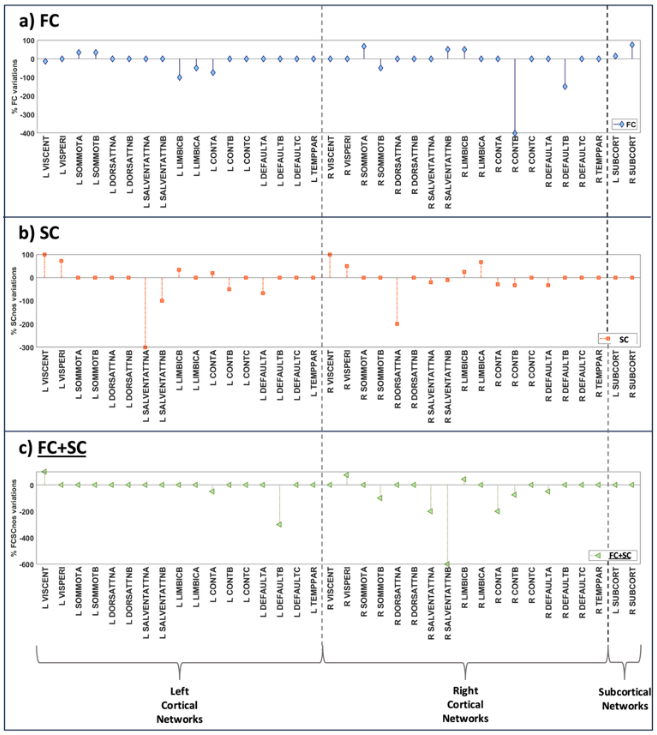

Supplementary Figure 8: Transfer learning impact was evaluated in terms of ND variations. For each network and each modality, ND variations are evaluated and displayed. Panel a) refers to FC-based variations, Panel b) refers to SC-based variations, and Panel c) refers to FC+SC-based variations. VisCent = Visual Central network; VisPeri = Visual Peripheral network; SomMotA = Somatomotor-A network; SomMotB = Somatomotor-B network; DorsAttnA = Dorsal Attention-A network; DorsAttnB = Dorsal Attention-B network; SalVentAttnA = Salience/Ventral Attention-A network; SalVentAttB = Salience/Ventral Attention-B network; LimbicB = Limbic-B network; LimbicA = Limbic-A network; ControlA = Control-A network; ControlB = Control-B network; ControlC = Control-C network; DefaultA = Default-A network; DefaultB = Default-B network; DefaultC = Default-C network; TempPar = Temporal Parietal network; Subcort = Subcortical network

2.4 Disentangling Feature Space

Using t-SNE for visualization and k-means for qualitative partitioning (see Supplementary Fig. 9, panel a), five partitions were observed in the healthy dataset. Varimax-rotated PCA loadings for functional connectivity (FC) and structural connectivity (SC) are shown in Supplementary Fig. 10 (panel a; FC on the left and SC on the right). With an absolute loading threshold of 0.12, the functional connectivity (FC) representations highlighted similar networks across most partitions (groups 1, 2, 3, and 5), whereas partition 4 emphasized the left and right Somatomotor-A and Somatomotor-B networks. Homotopic network pairs were consistently prominent. For the SC dataset, the varimax loadings remained stable across partitions and predominantly represented the left Somatomotor areas.

The same descriptive procedure was applied to the oncological dataset, where four partitions were observed (see Supplementary Fig. 9, panel b). FC varimax loadings indicated a different organization than the healthy data (see Supplementary Fig. 10, panel b; FC on the left). Again, homotopic networks were prominent. Visually, partition 4 appeared similar to partition 2. SC varimax results (see Supplementary Fig. 10, panel b; SC on the right) showed comparable loadings for partitions 2 and 4. Across partitions, the networks that contributed most to the representation in FC and SC were not necessarily concurrent.

Importantly, features labelled as “most informative” by Varimax (i.e., those with the largest absolute rotated loadings) did not correspond to networks flagged as abnormal by the anomaly detection framework. This reflects the distinct aims of these analyses: variance representation versus deviation from a healthy reconstruction. Healthy data exhibited relatively consistent parcel patterns across partitions, particularly for SC, whereas the oncological dataset displayed more variation among partitions. Surface views and Varimax-highlighted parcels are provided in Supplementary Figures 11 and 12. Supplementary Figure 11 shows healthy data, and Supplementary Figure 12 shows oncological data. Panel A shows FC, and Panel B shows SC. With a preliminary analysis no links were identified between the patients’ subgrouping and clinical or demographical information. This analysis is exploratory in nature and intended mainly to describe the latent variance structure. As such, it is not used for any inference on the pathology and does not currently define any clinically meaningful subgroups.

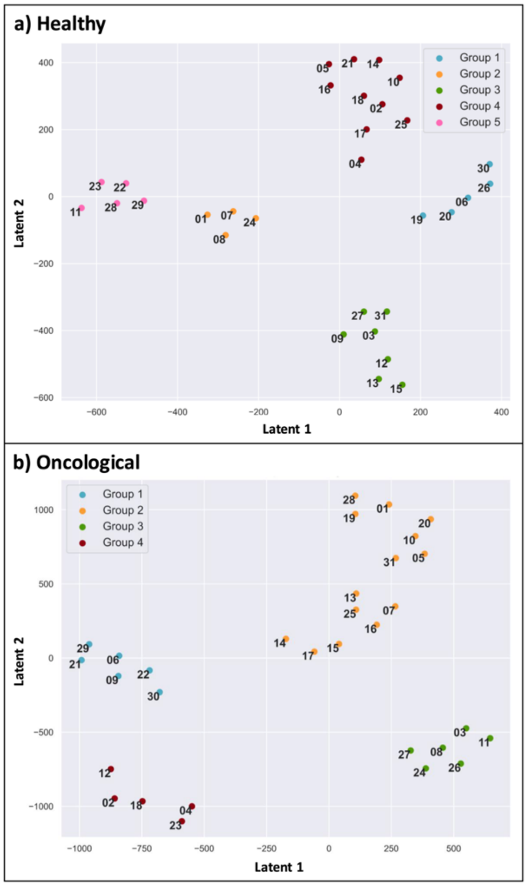

Supplementary Figure 9: tSNE visual representation of latent space features and k-means clustering results. Panel a): tSNE representation of latent space features derived from HCP healthy data. K-means provided 5 clusters that are displayed in the legend. Panel b): tSNE representation of latent space features derived from oncological test patients. K-means provided 4 clusters that are displayed in the legend.

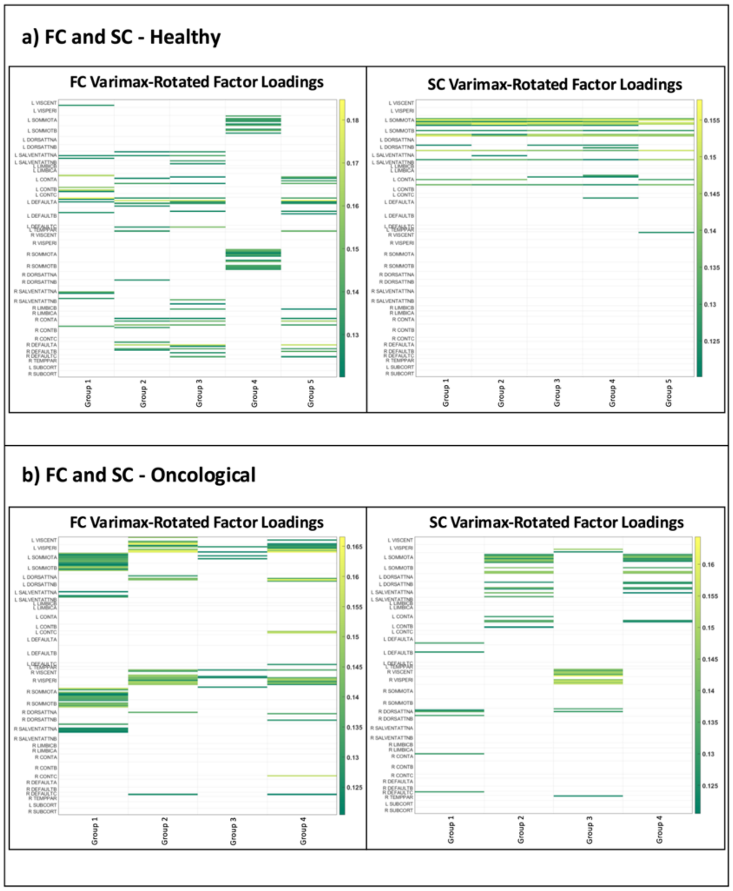

Supplementary Figure 10: Varimax rotational technique outcomes applied on the first three principal components of a principal components analysis. On the right side there are results obtained from FC matrices, on the left side there are results obtained from SC matrices. Each column refers to each group individualized by the tSNE and k-means procedure. Panel a): varimax results for HCP healthy data. Panel b): varimax results for oncological data. VisCent = Visual Central network; VisPeri = Visual Peripheral network; SomMotA = Somatomotor-A network; SomMotB = Somatomotor-B network; DorsAttnA = Dorsal Attention-A network; DorsAttnB = Dorsal Attention-B network; SalVentAttnA = Salience/Ventral Attention-A network; SalVentAttB = Salience/Ventral Attention-B network; LimbicB = Limbic-B network; LimbicA = Limbic-A network; ControlA = Control-A network; ControlB = Control-B network; ControlC = Control-C network; DefaultA = Default-A network; DefaultB = Default-B network; DefaultC = Default-C network; TempPar = Temporal Parietal network; Subcort = Subcortical network.

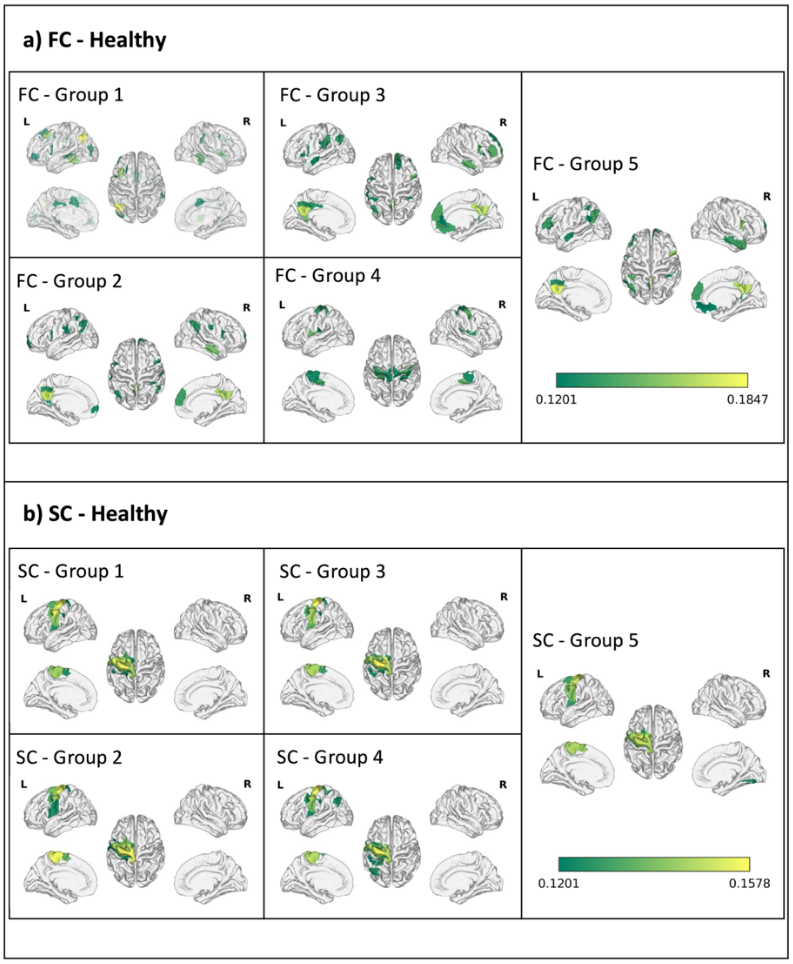

Supplementary Figure 11: Surface brain representation highlighting parcels enhanced by the varimax procedure on HCP subjects. In Panel a) there are results obtained from FC matrices, in Panel b) there are results obtained from SC matrices. Each box outlines a different group.

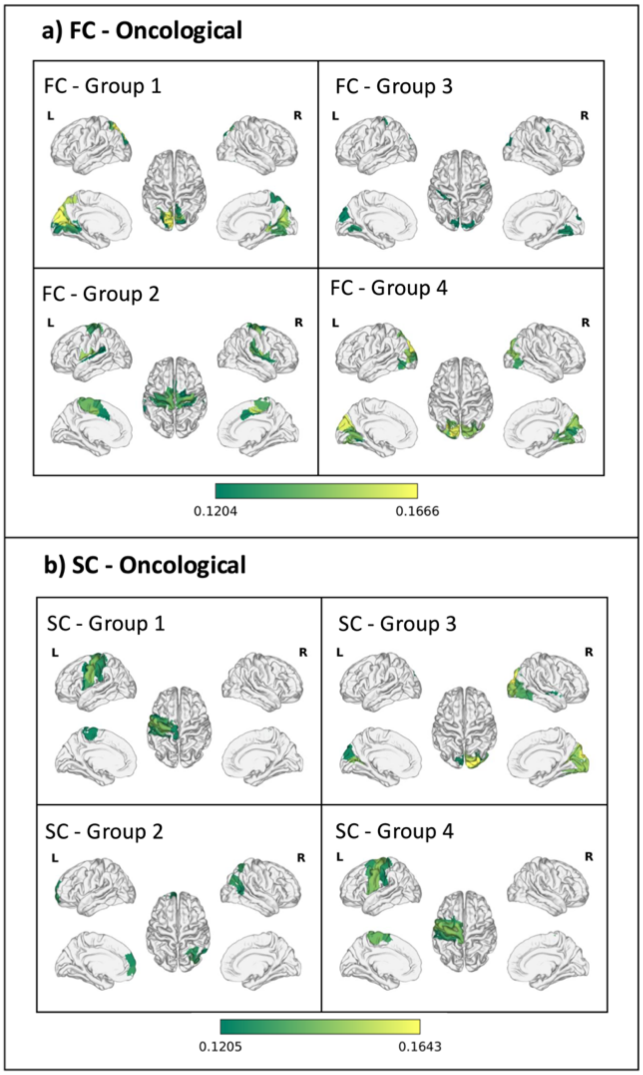

Supplementary Figure 12: Surface brain representation highlighting parcels enhanced by the varimax procedure on oncological subjects. In Panel a) there are results obtained from FC matrices, in Panel b) there are results obtained from SC matrices. Each box outlines a different group.

References:

1. Louis DN, Perry A, Reifenberger G, et al. The 2016 World Health Organization Classification of Tumors of the Central Nervous System: a summary. *Acta Neuropathol.Springer Verlag*. 2016;131(6):803-820. doi:10.1007/s00401-016-1545-1
2. Zhang H, Schneider T, Wheeler-Kingshott CA, Alexander DC. NODDI: Practical in vivo neurite orientation dispersion and density imaging of the human brain. *Neuroimage*. 2012;61(4):1000-1016. doi:10.1016/j.neuroimage.2012.03.072
3. Harms MP, Somerville LH, Ances BM, et al. Extending the Human Connectome Project across ages: Imaging protocols for the Lifespan Development and Aging projects. *Neuroimage*. 2018;183:972-984. doi:10.1016/j.neuroimage.2018.09.060
4. Tustison NJ, Avants BB, Cook PA, et al. N4ITK: improved N3 bias correction. *IEEE Trans Med Imaging*. 2010;29(6):1310-1320. doi:10.1109/TMI.2010.2046908

5. Doshi J, Erus G, Ou Y, Gaonkar B, Davatzikos C. Multi-atlas skull-stripping. *Acad Radiol*. 2013;20(12):1566-1576.
6. Ashburner J, Friston KJ. Unified segmentation. *Neuroimage*. 2005;26(3):839-851. doi:10.1016/j.neuroimage.2005.02.018
7. Fonov V, Evans AC, Botteron K, Almli CR, McKinstry RC, Collins DL. Unbiased average age-appropriate atlases for pediatric studies. *Neuroimage*. 2011;54(1):313-327. doi:10.1016/j.neuroimage.2010.07.033
8. Avants BB, Tustison NJ, Song G, Cook PA, Klein A, Gee JC. A reproducible evaluation of ANTs similarity metric performance in brain image registration. *Neuroimage*. 2011;54(3):2033-2044. doi:10.1016/j.neuroimage.2010.09.025
9. Andersen SM, Rapcsak SZ, Beeson PM. Cost function masking during normalization of brains with focal lesions: still a necessity? *Neuroimage*. 2010;53(1):78-84.
10. Tournier JD, Mori S, Leemans A. Diffusion tensor imaging and beyond. *Magn Reson Med.John Wiley and Sons Inc*. 2011;65(6):1532-1556. doi:10.1002/mrm.22924
11. Bastiani M, Cottaar M, Fitzgibbon SP, et al. Automated quality control for within and between studies diffusion MRI data using a non-parametric framework for movement and distortion correction. *Neuroimage*. 2019;184:801-812. doi:10.1016/j.neuroimage.2018.09.073
12. Tournier JD, Smith R, Raffelt D, et al. MRtrix3: A fast, flexible and open software framework for medical image processing and visualisation. *Neuroimage*. 2019;202:116137. doi:10.1016/j.neuroimage.2019.116137
13. Veraart J, Novikov DS, Christiaens D, Ades-Aron B, Sijbers J, Fieremans E. Denoising of diffusion MRI using random matrix theory. *Neuroimage*. 2016;142:394-406. doi:10.1016/j.neuroimage.2016.08.016
14. Andersson JLR, Sotiropoulos SN. An integrated approach to correction for off-resonance effects and subject movement in diffusion MR imaging. *Neuroimage*. 2016;125:1063-1078. doi:10.1016/j.neuroimage.2015.10.019
15. Andersson JLR, Skare S, Ashburner J. How to correct susceptibility distortions in spin-echo echo-planar images: Application to diffusion tensor imaging. *Neuroimage*. 2003;20(2):870-888. doi:10.1016/S1053-8119(03)00336-7
16. Avants BB, Tustison NJ, Song G, Cook PA, Klein A, Gee JC. A reproducible evaluation of ANTs similarity metric performance in brain image registration. *Neuroimage*. 2011;54(3):2033-2044. doi:10.1016/j.neuroimage.2010.09.025
17. Smith SM, Jenkinson M, Woolrich MW, et al. Advances in functional and structural MR image analysis and implementation as FSL. In: *NeuroImage*. Vol 23. 2004. doi:10.1016/j.neuroimage.2004.07.051
18. Jeurissen B, Tournier JD, Dhollander T, Connelly A, Sijbers J. Multi-tissue constrained spherical deconvolution for improved analysis of multi-shell diffusion MRI data. *Neuroimage*. 2014;103:411-426. doi:10.1016/j.neuroimage.2014.07.061
19. Smith RE, Tournier JD, Calamante F, Connelly A. Anatomically-constrained tractography: Improved diffusion MRI streamlines tractography through effective use of anatomical information. *Neuroimage*. 2012;62(3):1924-1938. doi:10.1016/j.neuroimage.2012.06.005
20. Tournier JD, Calamante F, Connelly A. Improved probabilistic streamlines tractography by 2nd order integration over fibre orientation distributions. *Proceedings of the International Society for Magnetic Resonance in Medicine*. Published online 2010:1670.
21. Smith RE, Tournier JD, Calamante F, Connelly A. SIFT: Spherical-deconvolution informed filtering of tractograms. *Neuroimage*. 2013;67:298-312. doi:10.1016/j.neuroimage.2012.11.049
22. Smith SM, Jenkinson M, Woolrich MW, et al. Advances in functional and structural MR image analysis and implementation as FSL. In: *NeuroImage*. Vol 23. 2004. doi:10.1016/j.neuroimage.2004.07.051
23. Greve DN, Fischl B. Accurate and robust brain image alignment using boundary-based registration. *Neuroimage*. 2009;48(1):63-72. doi:10.1016/j.neuroimage.2009.06.060

24. Salimi-Khorshidi G, Douaud G, Beckmann CF, Glasser MF, Griffanti L, Smith SM. Automatic denoising of functional MRI data: Combining independent component analysis and hierarchical fusion of classifiers. *Neuroimage*. 2014;90:449-468. doi:10.1016/j.neuroimage.2013.11.046
25. Griffanti L, Salimi-Khorshidi G, Beckmann CF, et al. ICA-based artefact removal and accelerated fMRI acquisition for improved resting state network imaging. *Neuroimage*. 2014;95:232-247. doi:10.1016/j.neuroimage.2014.03.034
26. Damaraju E, Allen EA, Belger A, et al. Dynamic functional connectivity analysis reveals transient states of dysconnectivity in schizophrenia. *Neuroimage Clin*. 2014;5:298-308. doi:10.1016/j.nicl.2014.07.003
27. Silvestri E, Moretto M, Facchini S, et al. Widespread cortical functional disconnection in gliomas: an individual network mapping approach. *Brain Commun*. 2022;4(2). doi:10.1093/braincomms/fcac082
28. Jo HJ, Gotts SJ, Reynolds RC, et al. Effective preprocessing procedures virtually eliminate distance-dependent motion artifacts in resting state FMRI. *J Appl Math*. 2013;2013. doi:10.1155/2013/935154
29. Power JD, Barnes KA, Snyder AZ, Schlaggar BL, Petersen SE. Spurious but systematic correlations in functional connectivity MRI networks arise from subject motion. *Neuroimage*. 2012;59(3):2142-2154. doi:10.1016/j.neuroimage.2011.10.018
30. Sara U, Akter M, Uddin MS. Image Quality Assessment through FSIM, SSIM, MSE and PSNR—A Comparative Study. *Journal of Computer and Communications*. 2019;07(03):8-18. doi:10.4236/jcc.2019.73002
31. Wang Z, Bovik AC, Sheikh HR, Simoncelli EP. Image quality assessment: From error visibility to structural similarity. *IEEE Transactions on Image Processing*. 2004;13(4):600-612. doi:10.1109/TIP.2003.819861
32. Wang Z, Bovik AC. Mean squared error: Lot it or leave it? A new look at signal fidelity measures. *IEEE Signal Process Mag*. 2009;26(1):98-117. doi:10.1109/MSP.2008.930649
33. Bovik A, Wang Z, Sheikh H. Structural Similarity Based Image Quality Assessment. In: 2005:225-241. doi:10.1201/9781420027822.ch7
34. Wang' Z, Simoncelli' ?, Bovik2 AC. *MULTI-SCALE STRUCTURAL SIMILARITY FOR IMAGE QUALITY ASSESSMENT*.
35. Weiss K, Khoshgoftaar TM, Wang DD. A survey of transfer learning. *J Big Data*. 2016;3(1). doi:10.1186/s40537-016-0043-6
36. Civier O, Sourty M, Calamante F. MFCSC: Novel method to calculate mismatch between functional and structural brain connectomes, and its application for detecting hemispheric functional specialisations. *Sci Rep*. 2023;13(1). doi:10.1038/s41598-022-17213-z
37. Van Der Maaten L, Hinton G. *Visualizing Data Using T-SNE*. Vol 9.; 2008.
